# Multisite Phosphorylation of NuMA-Related LIN-5 Controls Mitotic Spindle Positioning in *C*. *elegans*

**DOI:** 10.1371/journal.pgen.1006291

**Published:** 2016-10-06

**Authors:** Vincent Portegijs, Lars-Eric Fielmich, Matilde Galli, Ruben Schmidt, Javier Muñoz, Tim van Mourik, Anna Akhmanova, Albert J. R. Heck, Mike Boxem, Sander van den Heuvel

**Affiliations:** 1 Developmental Biology, Department of Biology, Faculty of Science, Utrecht University, Utrecht, The Netherlands; 2 Cell Biology, Department of Biology, Faculty of Science, Utrecht University, Utrecht, The Netherlands; 3 Biomolecular Mass Spectrometry and Proteomics, Bijvoet Center for Biomolecular Research and Utrecht Institute for Pharmaceutical Sciences, Utrecht University, Utrecht, The Netherlands; The University of North Carolina at Chapel Hill, UNITED STATES

## Abstract

During cell division, the mitotic spindle segregates replicated chromosomes to opposite poles of the cell, while the position of the spindle determines the plane of cleavage. Spindle positioning and chromosome segregation depend on pulling forces on microtubules extending from the centrosomes to the cell cortex. Critical in pulling force generation is the cortical anchoring of cytoplasmic dynein by a conserved ternary complex of Gα, GPR-1/2, and LIN-5 proteins in *C*. *elegans* (Gα–LGN–NuMA in mammals). Previously, we showed that the polarity kinase PKC-3 phosphorylates LIN-5 to control spindle positioning in early *C*. *elegans* embryos. Here, we investigate whether additional LIN-5 phosphorylations regulate cortical pulling forces, making use of targeted alteration of *in vivo* phosphorylated residues by CRISPR/Cas9-mediated genetic engineering. Four distinct *in vivo* phosphorylated LIN-5 residues were found to have critical functions in spindle positioning. Two of these residues form part of a 30 amino acid binding site for GPR-1, which we identified by reverse two-hybrid screening. We provide evidence for a dual-kinase mechanism, involving GSK3 phosphorylation of S659 followed by phosphorylation of S662 by casein kinase 1. These LIN-5 phosphorylations promote LIN-5–GPR-1/2 interaction and contribute to cortical pulling forces. The other two critical residues, T168 and T181, form part of a cyclin-dependent kinase consensus site and are phosphorylated by CDK1-cyclin B *in vitro*. We applied a novel strategy to characterize early embryonic defects in lethal T168,T181 knockin substitution mutants, and provide evidence for sequential LIN-5 N-terminal phosphorylation and dephosphorylation in dynein recruitment. Our data support that phosphorylation of multiple LIN-5 domains by different kinases contributes to a mechanism for spatiotemporal control of spindle positioning and chromosome segregation.

## Author Summary

Protein kinases control biological processes by phosphorylating specific amino acids of substrate proteins. It remains a major challenge to identify which phosphorylation events are critical *in vivo* and how phosphorylation affects protein function. Recent developments in CRISPR/Cas9-based genetic engineering make it possible to substitute individual amino acids, which allows investigating the role of single and multi-site phosphorylation of substrates *in vivo*. Here, we focus on an intensively phosphorylated cell division protein, LIN-5^NuMA^. *C*. *elegans* LIN-5 participates in chromosome segregation and is essential for positioning the spindle and cell cleavage plane during asymmetric cell division. Previously, we demonstrated that the polarity kinase PKC-3 phosphorylates LIN-5 to inhibit its function. Our current analysis reveals four additional phosphorylated residues that are critical for LIN-5 function. Two of these residues contribute to the interaction of LIN-5 with its binding partner GPR-1/2, whereas the other two residues are critical in dynein motor recruitment by LIN-5. Together, our results reveal that multisite phosphorylation of LIN-5 is essential to ensure proper chromosome segregation and spindle positioning.

## Introduction

Animal development and tissue homeostasis depend critically on cell divisions that create cells with specific shapes and functions, in the right numbers and at the proper positions. The spindle apparatus plays a central role in the cell division process, as it segregates the chromosomes in mitosis and determines the plane of cell cleavage during cytokinesis [1–3]. Placement of the spindle in the cell center during division results in the formation of daughter cells of equal size, whereas off-center migration and spindle rotation allows the creation of differently sized daughter cells at specific locations. Moreover, the plane of cell cleavage determines whether polarized cells undergo symmetric or asymmetric cell division. Asymmetric cell divisions create cell diversity and allow maintenance of tissue-specific stem cells, by combining self-renewal with the generation of differentiating daughter cells (Reviews: [4,5]). Thus, tight control of the spindle function and position is needed to coordinate chromosome segregation with cleavage plane determination, which is essential for genetic stability, tissue integrity and stem cell maintenance in a wide variety of evolutionary contexts.

Pioneering studies in *Caenorhabditis elegans* and *Drosophila melanogaster* revealed that the position of the spindle responds to polarity cues during asymmetric cell division [1,2,4,5]. In *C*. *elegans*, anterior-posterior (A-P) polarity is established after fertilization of the oocyte. This involves re-distribution of specific partitioning-defective (PAR) proteins into two opposing domains of the cell cortex. The PDZ-domain proteins PAR-3 and PAR-6 form a complex with the PKC-3 aPKC polarity kinase and become restricted to the anterior half of the zygote, while the PAR-2 ring-finger protein and PAR-1 kinase occupy the posterior domain [6]. This A-P polarity guides the asymmetric localization of cytoplasmic determinants as well as the position of the mitotic spindle. During the first mitotic division, the spindle is positioned off-center, to instruct an asymmetric cell division that creates a larger anterior blastomere (AB) and smaller germline precursor cell (P1). Next, the spindle rotates by 90 degrees in P1, to instruct another asymmetric division with a cleavage plane perpendicular to the one of AB. These early divisions of the *C*. *elegans* embryo have served as an important model for studies of the coordinated regulation of cell polarity, fate determinant localization, and spindle positioning during asymmetric cell division.

**Fig 1:**
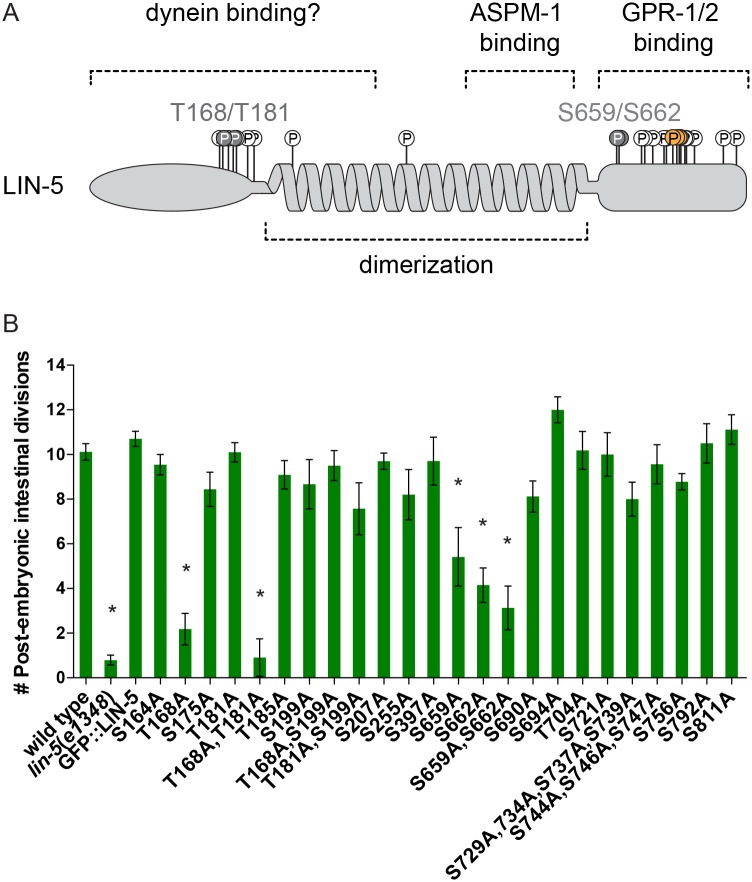
Phosphorylation of LIN-5 controls post-embryonic divisions in the intestine. **(A)** Overview of LIN-5 structure and binding domains. *In vivo* phosphorylated residues are indicated by open circles. Grey circles and numbers represent the phosphorylated amino acids essential for LIN-5 function. Yellow circles indicate previously identified residues phosphorylated by PKC-3 **(B)** Quantification of post-embryonic intestinal divisions in heterozygous *lin-5*(*e1348*) / *mIn1* (wild type), homozygous *lin-5*(*e1348*) animals and homozygous *lin-5*(*e1348*) animals expressing GFP-LIN-5 transgenes with alterations of the indicated phosphosite(s). n = 7–22 (average: 15). Error bars, s.e.m. * P < 0.01 compared to wild type, Unpaired Students T-test.

In addition, studies in *C*. *elegans* and *Drosophila* uncovered an evolutionarily conserved protein complex that mediates spindle positioning. This complex consists of the alpha subunit of a heterotrimeric G protein in association with the TPR/GoLoco protein GPR-1/2 and coiled-coil protein LIN-5 in *C*. *elegans* (Gα–Pins–Mud in *Drosophila*, Gα–LGN–NuMA in mammals) (Reviews: [1–6]). The GPR-1/2 GoLoco motifs interact with Gα-GDP [7], while the tetratricopeptide repeats (TPR) associate with the C-terminus of LIN-5 (Fig 1A). The ternary protein complex acts at the cell cortex in conjunction with cytoplasmic dynein and microtubule plus ends to generate microtubule pulling forces that promote chromosome segregation and position the spindle [8–12]. Based on results obtained for NuMA, an extended N-terminal domain of LIN-5 likely mediates interaction with the dynein motor complex [9]. It remains unclear how Gα–GPR-1/2–LIN-5 engages dynein and microtubule depolymerization in the generation of cortical pulling forces, and how pulling forces are temporally and spatially restricted.

Asymmetric positioning and rotation of the spindle result from imbalance in the pulling forces. It has long been known that the cortical polarity of the *C*. *elegans* zygote is fundamental for the spatial organization of pulling forces, creating a higher net force in the posterior than the anterior, which causes the spindle to move off center [13,14]. This is in part achieved through PKC-3 mediated phosphorylation of LIN-5, which inhibits anteriorly directed pulling forces [15]. Phosphorylation also appears to regulate cortical pulling forces in other systems. For example, phosphorylation by aPKC inhibits Pins/LGN localization to the apical cell membrane and promotes planar cell division of MDCK canine kidney cells during cyst formation [16]. Moreover, phosphorylation of NuMA by PLK1 and CDK1 has been implicated in the timing of chromosome segregation and positioning of the mitotic spindle in human cells [17,18]. In addition to spindle positioning, the Gα–GPR-1/2–LIN-5 complex is essential for chromosome segregation, in all cell divisions except for the first few embryonic divisions in *C*. *elegans* [19–21]. Phosphorylation is likely to play a key role in coordinating chromosome segregation and spindle positioning through spatiotemporal regulation of Gα–GPR-1/2–LIN-5 function.

Our previous studies identified extensive *in vivo* phosphorylation of LIN-5 in *C*. *elegans* embryos [15]. The function of the majority of these phosphorylations remained unknown. Here we apply a combination of techniques to determine which phosphorylations are critical for LIN-5 function. CRISPR/Cas9-mediated genetic engineering allowed us to introduce single codon alterations in the *C*. *elegans* genome, and to compare non-phosphorylatable and potentially phosphomimetic LIN-5 mutants. In addition to PKC-3, we found that the PAR-1 polarity kinase likely phosphorylates LIN-5 *in vivo*, but physiological consequences of this phosphorylation were not detected. Alanine substitution mutagenesis of *lin-5* transgenes pointed to four phosphorylated residues with critical functional contributions. Two of these residues form part of a 30 amino-acid domain of LIN-5 required for binding GPR-1/2. Phosphorylation of these residues promotes cortical pulling forces and GPR-1/2 localization *in vivo*, and appears to occur sequentially by GSK3 and casein kinase 1 (CK1). Moreover, we identified essential residues in the LIN-5 N-terminus that are phosphorylated by CDK1. Our data from extensive knockin replacement mutants are consistent with a mechanism involving sequential phosphorylation and dephosphorylation of the LIN-5 N-terminus in dynein recruitment to the meiotic spindle and cell cortex. Thus, a combination of phosphorylations by cell-cycle and polarity associated kinases likely underlies the spatiotemporal control of pulling forces in chromosome segregation and asymmetric cell division.

## Results

### Multiple *in vivo* phosphorylated residues are critical for LIN-5 function

Previously, we described that at least 25 residues of LIN-5 are phosphorylated *in vivo* (Fig 1A) [15]. To acquire insight in which phosphorylations are functionally relevant, we replaced each phosphorylated serine or threonine with an alanine residue that cannot be phosphorylated. The relevant codon alterations were introduced in a cloned genomic *lin-5* DNA fragment and subsequently tested for functionally complementing the *lin-5*(*e1348*) null mutation *in vivo* [19]. In the presence of maternal product, *lin-5*(*e1348*) mutants fail to undergo chromosome segregation during postembryonic divisions and continue abortive mitoses [19–21]. Transgenes containing wild type *lin-5* or *gfp*::*lin-5* coding sequences restored post-embryonic cell divisions in *lin-5*(*e1348*) null mutants (Fig 1B). However, these *lin-5* transgenes appeared susceptible to germline and somatic silencing, as reliable rescue and GFP-LIN-5 expression was observed only in the F1 generation. Hence, we examined transgenic F1 animals, focusing on vulval development and nuclear divisions in the intestine as a quantitative measure for LIN-5 function (Fig 1B).

Alanine substitutions of threonine 168, serine 659, and serine 662 were the only single amino acid changes that significantly compromised LIN-5 function *in vivo*. The T168A mutation had the strongest effect and almost completely eliminated the ability to restore intestinal divisions in *lin-5*(*e1348*) null mutants (Fig 1B). Interestingly, this strong effect was specific for the intestine: LIN-5^T168A^ expression allowed *lin-5* mutants to develop a normal vulva (S1 Fig). T168 forms part of an ideal consensus phosphorylation site (S/T*-P-x-K/R) for the mitotic cyclin-dependent kinase 1 (CDK-1) [22]. CDK-1 is likely to regulate LIN-5, as multiple CDK-1 consensus sites are present in the LIN-5 N- and C-terminus, and CDK1 phosphoregulation of the NuMA C-terminus has been reported [18,23]. We generated double alanine substitutions of T168 in combination with T181 or S199, two nearby candidate residues for CDK-1 phosphorylation. Strikingly, the transgene encoding LIN-5[T168A,T181A], but not LIN-5[T168A,S199A], completely failed to rescue intestinal mitoses and vulva formation in *lin-5*(*e1348*) mutants (Fig 1B and S1 Fig). Because phosphorylation of T181 by itself was not essential for post-embryonic divisions, T168 and T181 phosphorylations likely cooperate to control LIN-5 function.

The individual and combined S659A and S662A substitutions (LIN-5[S659A,S662A]) also reduced *lin-5*(*e1348*) complementation. By contrast, simultaneous alanine substitutions of serines 729, 734, 737, and 739 did not prevent LIN-5 function (Fig 1B and S1 Fig). In agreement with the latter result, PKC-3 (aPKC) phosphorylation of these residues inhibits LIN-5 function and is not required for cell division [15]. Our alanine-substitution experiments indicate that in addition to spatiotemporal regulation of LIN-5 by PKC-3, phosphorylation of LIN-5 residues in the dynein-interacting N-terminus and GPR-1/2 binding C-terminus may contribute to LIN-5 regulation *in vivo*.

### *In vitro* kinase assays reveal candidate LIN-5 kinases

To determine whether CDK1 is indeed able to phosphorylate T168 and T181 of LIN-5, we performed *in vitro* kinase assays with recombinant GST-LIN-5 expressed in *E*. *coli* as a substrate. Indeed, immunopurified human CDK1/cyclin B phosphorylated GST-LIN-5, but not GST alone (S2A and S2B Fig). Analysis of *in vitro* phosphorylated GST-LIN-5 by mass spectrometry revealed extensive phosphorylation of T168, T181, and S744 of LIN-5 (S2B Fig). Additionally, peptides containing phosphorylated T704 and S756 were also found, and some other phosphopeptides less frequently. Taken together, CDK1/cyclin B phosphorylates LIN-5 *in vitro* at multiple sites including T168 and T181, and phosphorylation of T168 and T181 *in vivo* appears to be required for LIN-5 function.

**Fig 2:**
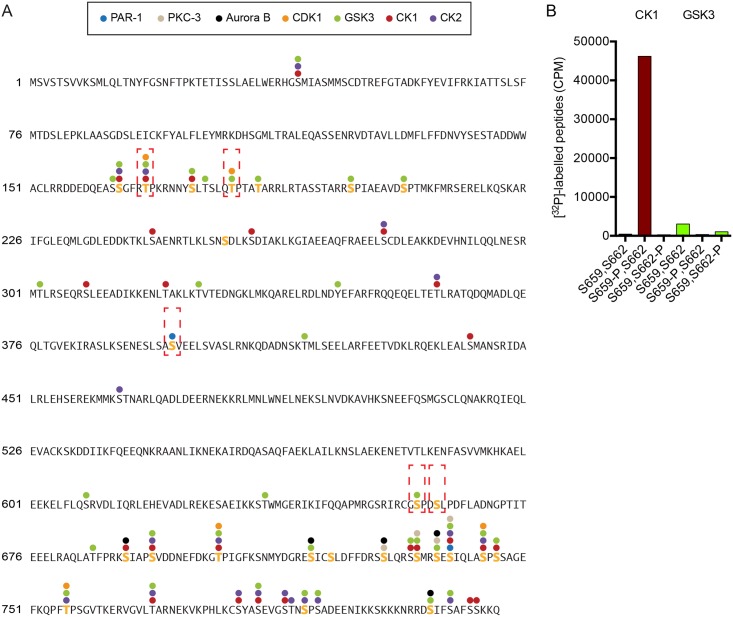
LIN-5 phosphorylation by cell cycle and polarity kinases *in vitro*. **(A)** Graphic overview of mass spectrometry analysis of *in vitro* kinase assays, revealing multiple target residues in LIN-5 phosphorylated by PAR-1, PKC-3, CK1, CK2, GSK3, CDK1 and Aurora B kinase. Yellow residues indicate *in vivo* phosphorylated residues, brackets indicate essential residues identified in complementation assay. **(B)** Radioactive counts (CPM) of *in vitro* kinase assays with CK1 and GSK3 on a synthetic LIN-5 peptide 654–670 with or without synthetic incorporation of phosphorylated amino acids S659 or S662.

In contrast to T168 and T181, residues S659 and S662 are not part of apparent consensus phosphorylation sites. In our previous *in vivo* mass-spectrometry data, the S659,S662 double phosphorylated peptides were abundant, while the corresponding unphosphorylated peptides were not detected [15]. This may indicate that S659 and S662 are constitutively phosphorylated in early embryos. To gain insight in which kinases may be involved, we examined LIN-5 phosphorylation *in vitro* with a series of polarity and cell cycle kinases, followed by mass spectrometry analyses. This revealed several residues that were phosphorylated by multiple kinases *in vitro* (Fig 2A). In striking contrast, S659 was only phosphorylated by GSK3, and none of the tested kinases phosphorylated S662 (Fig 2A). We considered several potential explanations for this lack of phosphorylation: the responsible kinase(s) may not have been included in the assays, residue S662 may not be accessible in the recombinant protein, or S662 phosphorylation may require a priming event. To test the latter possibility, we performed *in vitro* kinase assays with synthetic peptides that contain the S659 and S662 residues, either unphosphorylated or phosphorylated at one of the positions. Testing several kinases, we found that casein kinase 1 (CK1) efficiently phosphorylates S662, but only when the peptide contained a phosphorylated S659 residue (Fig 2B). As for the full length protein, only GSK3 phosphorylated S659 in the unphosphorylated peptide. Based on the combined *in vitro* data, we propose that GSK3 phosphorylation of residue S659 is a priming reaction for CK1 phosphorylation of S662. Highly similar phosphorylation has been reported for the Wnt/Frizzled co-receptor LRP6, with GSK3 priming for CK1 phosphorylation at similar sites [24,25].

In addition to CDK1, GSK3 and CK1 phosphorylation, our analyses revealed phosphorylation of LIN-5 by the polarity kinase PAR-1. While several phosphopeptides were detected, some were rare and the quantitative software program MaxQuant only recognized the S397 and S739 LIN-5 residues as *in vitro* phosphorylated by PAR-1 (Fig 2A). S397 is located in the LIN-5 coiled coil region and its phosphorylation was previously observed in embryos (Fig 2A) [15]. However, our previous *in vivo* analysis failed to identify LIN-5 phosphorylations that were diminished after *par-1* RNAi [15]. Re-evaluation of the quantitative mass spectrometry data revealed that, although masked by an abundant unrelated peptide, the ratio between the phosphorylated and unphosphorylated S397 peptide was severely reduced in *par-1(RNAi)* embryos compared to control RNAi embryos (S3 Fig). In contrast, S739 phosphorylation was not significantly affected by *par-1* knockdown *in vivo* [15]. Taken together, we identified multiple phosphorylated LIN-5 residues as well as candidate kinases that could be important in the regulation of LIN-5 function. In addition to four adjoining residues phosphorylated by PKC-3 in the C-terminus, T168 and T181 may be phosphorylated by CDK-1, S397 by PAR-1, and S659 by GSK-3, to prime phosphorylation of S662 by CK1.

### A 30 amino acid LIN-5 domain that includes S659,S662 mediates GPR-1/2 interaction

**Fig 3:**
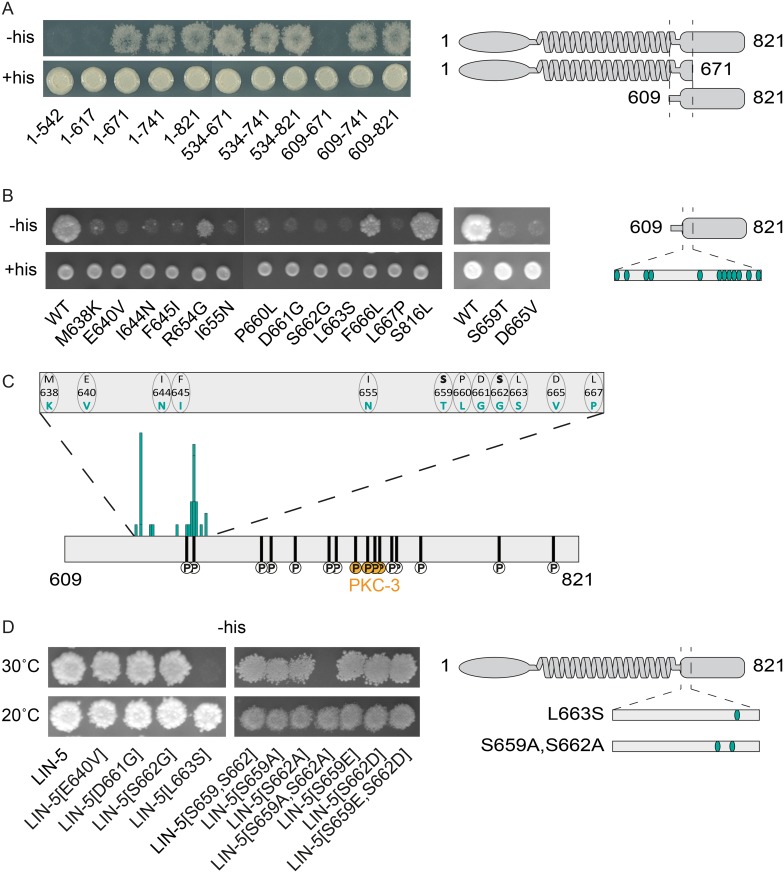
Yeast two-hybrid assays identify critical residues in LIN-5 for GPR-1 interaction. **(A)** Yeast two-hybrid analysis of DB::GPR-1 (bait) interaction with AD::LIN-5 (prey) fragments of varying sizes on Sc (synthetic complete) −Leu−Trp (+His, control) and Sc −Leu−Trp−His + 3-AT (-His, selection) plates. **(B)** Yeast two-hybrid analysis of DB::GPR-1 (bait) interaction with AD::LIN-5 (prey) fragments containing single amino acid changes on Sc −Leu−Trp and Sc −Leu−Trp−His + 3-AT plates. **(C)** Graphical representation of LIN-5 residues required for GPR-1 binding. Size of bars indicates frequency of found mutations, with every bar representing a different amino acid change. **(D)** Yeast two-hybrid analysis of DB::GPR-1 (bait) interaction with AD::LIN-5 (prey) full length containing single amino acid changes on Sc −Leu−Trp−His + 3-AT plates at 20°C and 30°C.

Both S659,S662 and the four residues phosphorylated by PKC-3 are located in the LIN-5 C-terminus which mediates GPR-1/2 binding [15,26]. As phosphorylation could affect GPR-1/2 association, we wanted to define which LIN-5 residues are critical for GPR-1/2 binding. Testing deletion constructs in yeast two-hybrid assays confirmed that the LIN-5 C-terminal region is sufficient for GPR-1 association. GPR-1 interaction was observed for all truncated LIN-5 proteins except for those with deletions in the 609–671 amino acid region (Fig 3A). At the same time, including only the 609–671 LIN-5 fragment did not allow growth in this assay, possibly due to an inability of this short fragment to fold properly in yeast (Fig 3A). The essential 609–671 region does not contain serine 729, 734, 737, and 739 phosphorylated by PKC-3 *in vivo*, in agreement with our previous conclusion that PKC-3 phosphorylation of LIN-5 does not prevent interaction with GPR-1/2 [15].

To identify specific LIN-5 amino acids required for GPR-1/2 interaction, we performed “reverse yeast two-hybrid screening”. This method selects mutations that disrupt bait-prey protein interactions, making use of *URA3*-mediated conversion of 5-fluoroorotic acid (5-FOA) to a toxic product [27]. The normal interaction between LIN-5 and GPR-1 leads to GAL4-controlled *URA3* expression in yeast two-hybrid assays, and causes cell death in the presence of 5-FOA. Thus, following mutagenesis of one of the binding partners, interaction-deficient alleles can be recovered from 5-FOA-resistant colonies [28]. We used PCR-based random mutagenesis of LIN-5 prey fragments (amino acids 609–821), and isolated 163 5-FOA resistant yeast colonies in a reverse yeast two-hybrid screen (for details see Materials and Methods, S4A Fig). 89 colonies contained a single missense mutation in the LIN-5 coding sequences, together changing 15 different amino acids. Substitutions of 12 of these 15 individual amino acids caused loss of GPR-1 interaction again in the re-test (Fig 3B and 3C). The 12 affected residues were all located between amino acids 638–667 of LIN-5. Importantly, the interaction-defective alleles included missense mutations of the phosphorylated residues S659 and S662. In fact, S662 was found altered to glycine, cysteine and asparagine (S4B Fig). These data indicate that a 30 amino acid stretch in the LIN-5 C-terminal region, which includes the *in vivo* phosphorylated S659 and S662 residues, mediates the interaction with GPR-1.

Following up on the interaction defective alleles, we noticed that the effect of missense mutations was substantially reduced when tested in the context of full length LIN-5, compared to the C-terminus only. Western blot analysis did not reveal substantial differences in protein levels compared to wild type (S5A Fig). The LIN-5 coiled-coil region promotes dimerization and is thereby expected to increase GPR-1 binding avidity. Only one of the four most frequently identified mutations, L663S, also interfered with full length LIN-5 binding to GPR-1 (Fig 3D, left panel). However, at a reduced temperature (20°C), this leucine 663 to serine (LIN-5[L663S]) mutation still allowed growth on selective media, indicating that GPR-1 interaction is not completely abolished. We also tested S659 and S662 phosphorylation-site mutants in the context of full length LIN-5. While the single mutations had little effect on GPR-1 binding, replacement of both serine 659 and 662 by alanine reduced GPR-1 interaction in yeast, as detected by lack of growth on -His plates at 30°C (Fig 3D, right panel). Phosphomimetic substitutions (S to D or E) of S659, S662, or both, did not reduce interaction (Fig 3D, right panel). These results are consistent with phosphorylation of S659 and S662 contributing to GPR-1/2 binding, and taking place in yeast as well as *C*. *elegans*. Taken together, our forward and reverse yeast two-hybrid assays identified LIN-5 residues that appear to mediate interaction with GPR-1/2, which are located within a 30 amino acid C-terminal domain. This includes S659 and S662, of which the phosphorylation *in vivo* likely contributes to GPR-1/2 binding.

### Examining LIN-5 phosphoregulation *in vivo* by CRISPR/Cas9-mediated genome engineering

**Fig 4:**
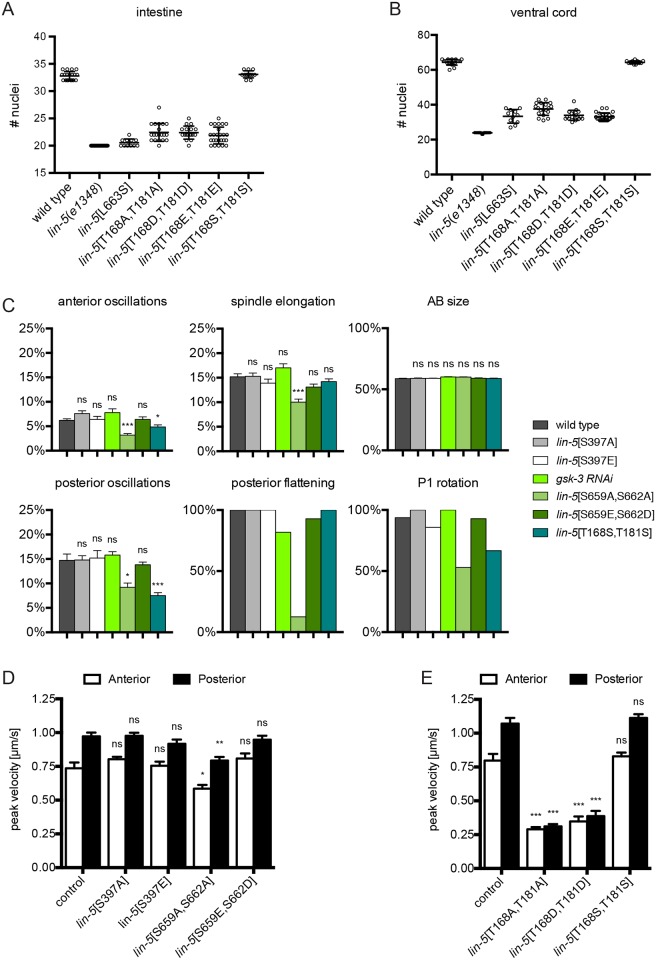
*In vivo* phenotypical analysis shows developmental defects in LIN-5 phosphomutants. **(A)** Quantification of intestinal nuclei following propidium iodide staining in homozygous LIN-5 phosphorylation mutants compared to wild type and *lin-5*(*e1348*) null animals. Null mutants are expected to contain 20 nuclei, wild type animals 32–34 nuclei. Individual values are plotted, n>11. **(B)** Quantification of P-cells and juvenile motor neurons in the ventral cord region P2-P10 in propidium iodide stained homozygous LIN-5 phosphorylation mutants compared to wild type and *lin-5*(*e1348*) null animals. Nuclei were counted in late larval stages of asynchronous populations of worms, with null mutants expected to have 24 nuclei (9 P cells and 15 juvenile motor neurons) and wild type animals 65 nuclei. Individual values are plotted, n>11. **(C)** Analysis of hallmarks of the first 2 embryonic divisions in LIN-5 phosphorylation mutant, based on time-lapse DIC microscopy. Oscillations are plotted as percentage of embryo height, elongation and AB size as a percentage of embryo width, flattening and rotation as a total fraction of analyzed embryos. **(D, E)** Mean peak velocities (μm s−1) of anterior and posterior spindle poles measured in a 12.5 s time frame after spindle severing in one-cell embryos of the indicated genotypes, cultured at 25°C (D) or 20°C (E). Error bars: s.e.m. Statistical analyses: Unpaired Welch Students T-test,*P < 0.01 compared to wild type, ** P < 0.001 compared to wild type, *** P < 0.0001 compared to wild type, ns not significant.

We used CRISPR/Cas9-mediated gene targeting to engineer *lin-5* alleles and examine the effects of amino acid substitutions *in vivo* [29–32]. First, we created the *lin-5*[L663S] mutation by introducing a single nucleotide alteration in the endogenous *lin-5* locus. This resulted in a typical *lin-5* loss-of-function phenotype, with homozygous sterile, thin and uncoordinated larvae that fail to undergo chromosome segregation but continue abortive mitoses [19,20]. We determined the number of nuclei in the intestine and ventral cord, following fixation and staining of DNA. In *lin-5*[L663S] mutants, both tissues contained severely reduced numbers of nuclei compared to the wild type, consistent with a failure to undergo chromosome segregation in most post-embryonic divisions (Fig 4A and 4B, S6A Fig). Thus, a single change of amino acid L663 in the GPR-1-binding motif of LIN-5 results in strong loss-of-*lin-5* function. This result confirms the power of reverse yeast two-hybrid screening in identifying amino acids that affect protein-protein interactions *in vivo* [27].

Next, we used genome engineering to alter the *in vivo* phosphorylated residues T168, T181, S397, S659 and S662. For each residue, we created a non-phosphorylatable alanine substitution allele, as well as one or more potentially phosphomimetic alleles that contain aspartic acid or glutamic acid at the relevant positions. Alteration of the PAR-1 phosphorylated S397 residue had no apparent effect. Homozygous S397A and S397E animals were viable and showed normal development. Even close examination of LIN-5-mediated processes did not reveal abnormalities (See below; Fig 4C and 4D, S6A and S6B Fig and S1 Video). Thus, although this phosphorylation occurs *in vivo*, it is by itself not a major determinant of LIN-5 function.

Compared to our transgene rescue experiments (Fig 1B and S1 Fig), the effect of S659 and S662 alanine substitution mutations in endogenous *lin-5* was quite mild. The *lin-5*[S659A,S662A] double mutant animals were viable, with only a slight reduction in intestinal nuclei number (S6A Fig), but displayed a significant increase in embryonic lethality (3.6±1.0% at 25°C, wild type 0.9±0.4%). The phosphomimetic *lin-5*[S659E,S662D] mutation did not cause embryonic lethality or larval defects, consistent with constitutive phosphorylation of these residues in early embryos (S6A Fig).

In stark contrast, alteration of the candidate CDK-1 phosphorylated residues in the N-terminus, threonine 168 and 181 to alanine (*lin-5*[T168A,T181A]), aspartic acid (*lin-5*[T168D,T181D]) or glutamic acid (*lin-5*[T168E,T181E]), all resulted in typical *lin-5* mutant offspring. Regardless of the mutant combination, homozygous animals derived from heterozygous parents developed into sterile, thin and uncoordinated larvae, and showed severely impaired cell division during larval development (Fig 4A and 4B). Importantly, substitution of threonine 168 and 181 with serine residues (*lin-5*[T168S, T181S]) did not lead to any detectable phenotype or defects in cell division (Fig 4A and 4B). These observations and the *in vivo* phosphorylation of T168 and T181 indicates that phosphoregulation of T168 and T181 is critical for LIN-5 function, in agreement with the results of the transgene rescue experiments (Fig 1B and S1 Fig). Together, our targeted genome alterations identified several individual amino acids that are required for the *in vivo* function of LIN-5, including phosphorylated residues in the N-terminus and residues in the GPR-1 binding domain.

### LIN-5 S659S662 phosphorylation contributes to cortical pulling forces

Additional characterizations of the non-phosphorylatable and phosphomimetic mutants revealed insight in the functional contribution of LIN-5 phosphorylation. As the contribution of S659 and S662 phosphorylation appeared quite subtle, we examined the spindle in early embryos with substitutions of these residues in detail. In the wild type, meiosis completes after fertilization and results in the formation of a haploid maternal pronucleus, which migrates to meet the paternal pronucleus in the posterior, after which the adjoined pronuclei and centrosomes migrate to the center, rotate and form a spindle along the long axis of the zygote [1,6] (S1 Video). Observations with differential interference contrast (DIC) microscopy showed that these events all occur normally in *lin-5*[S659A,S662A] and *lin-5*[S659E,S662D] mutants. Subsequently, in wild type embryos, the chromosomes become aligned at the metaphase plate and are segregated to opposite poles during anaphase. During spindle elongation, the posterior spindle pole oscillates extensively, while the anterior pole remains relatively steady. This coincides with spindle movement towards the posterior, and is followed by flattening of the posterior pole (S1 Video). Starting in anaphase, mutant embryos with non-phosphorylatable *lin-5*[S659A,S662A] deviated from the wild type, while *lin-5*[S659E,S662D] mutants showed no phenotype. Specifically, *lin-5*[S659A,S662A] mutants showed significantly dampened oscillation of both the anterior and posterior pole, reduced spindle elongation, and nearly absent flattening of the posterior spindle pole (Fig 4C and S6B Fig). Nevertheless, both non-phosphorylatable and phosphomimetic S659,S662 mutants underwent asymmetric division of the zygote as normal, which resulted in the formation of a larger anterior blastomere (AB) and smaller germline precursor cell (P1). The spindle normally rotates by 90 degrees prior to division of the P1 blastomere (S1 Video). This failed to occur or was incomplete in 47.1% of the *lin-5*[S659A,S662A] two-cell embryos, compared to 6.3% and 7.3% incomplete rotation scored in wild type and *lin-5*[S659E,S662D] mutant embryos, respectively (Fig 4C and S6B Fig). As protein levels were comparable to wild type (S5B Fig), these results suggest that cortical pulling forces are reduced in *lin-5*[S659A,S662A] mutants. Interestingly, this does not disrupt the asymmetry of the first division and has only a small effect on viability.

To determine cortical pulling forces more directly, we performed spindle severing assays with a UV laser beam [13]. Confirming our DIC analyses, the peak velocities of spindle pole movements were significantly reduced in *lin-5*[S659A,S662A] embryos (anterior pole 20.5%, posterior pole 18.4% reduced compared to wild type) (Fig 4D, S2 and S5 Videos). Similar experiments performed with *lin-5*[S659E,S662D] mutant embryos and PAR-1 phosphorylation site mutants (S397A and S397E) did not reveal significant divergence from the wild type (Fig 4D, S3, S4 and S6 Videos). These data support the conclusion that phosphorylation of S659 and S662 contributes to cortical pulling forces, both in the anterior and posterior, and thereby to spindle pole oscillation, spindle elongation, posterior pole flattening and spindle rotation in P1. Moreover, the finding that pulling forces, albeit reduced, remained asymmetric in *lin-5*[S659A, S662A] mutants explains why these mutants show normal asymmetry of the first division, and normal sizes of the AB and P1 blastomeres.

We wondered whether Wnt-signaling could locally control GSK-3 kinase activity to affect LIN-5 S659, S662 phosphorylation and asymmetric cell division. In the EMS blastomere of the 4-cell embryo, the spindle rotates from a left/right to anterior/posterior position to correctly specify and position the E and MS daughter cells [33]. This rotation is redundantly controlled by MES-1/SRC-1 and MOM-2/MOM-5 Wnt/Frizzled signaling pathways [34]. We examined whether the Wnt pathway contributes to EMS spindle rotation through phosphorylation of LIN-5[S659,S662]. Making use of a *mes-1*(*bn74*ts) mutant strain expressing GFP-β-tubulin, we observed normal spindle rotation in *lin-5*[S659A,S662A] mutant embryos, with only one of 13 embryos showing a tilted spindle angle in the EMS blastomere (S6C Fig). *mes-1*(*bn74*ts); *lin-5*[S659E,S662D] mutant embryos showed an occasional failed rotation or tilted spindle angle. In control *mes-1*(*bn74*ts); *gsk-3(RNAi)* mutants, the EMS spindle failed to rotate in 9/11 embryos (S6C Fig). This clear difference in phenotype shows that LIN-5 S659 phosphorylation is not the major contribution of GSK-3 in EMS spindle rotation. Asymmetric divisions of epithelial seam cells in the *C*. *elegans* epidermis also depend on a Wnt-β- catenin asymmetry pathway [35,36], and remained normal in *lin-5*[S659A,S662A] and *lin-5*[S659E,S662D] mutants. Thus, evidence for developmental regulation of LIN-5–GPR-1/2 interaction through Wnt-signaling was not obtained. Instead, absence of unphosphorylated S659,S662 peptides in our mass spectrometry analyses, and the wild type appearance of phosphomimetic mutants point to constitutive phosphorylation of the S659,S662 residues.

### GPR-1 fails to localize to the centrosome in LIN-5[S659A,S662A] mutants

**Fig 5:**
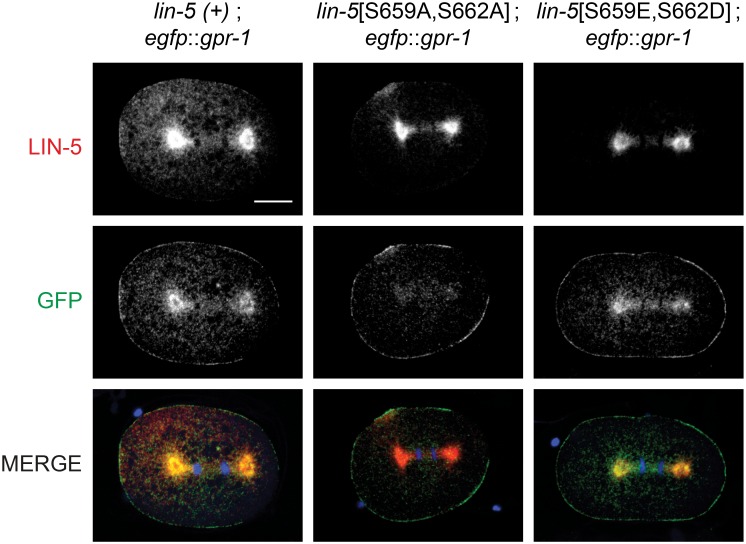
Localization of LIN-5 and GPR-1 in phosphorylation mutants. Immunohistochemical staining of embryos expressing wild type or phosphomutant *lin-5* and endogenously tagged *egfp*::*gpr-1*. Representative images of one-cell embryos in anaphase, stained with anti-LIN-5 (red) and anti-eGFP (green) antibodies, and DAPI to visualize DNA. All images taken with same exposure time, objective and magnification. Anterior to the left, scale bars 10 μm.

Our yeast two-hybrid data showed reduced interaction between LIN-5[S659A,S662A] and GPR-1 compared to wild type, which likely explains the reduced pulling forces observed *in vivo*. We examined whether the colocalization between LIN-5 and GPR-1 *in vivo* depends on LIN-5 phosphorylation. Hereto, we generated strains with *lin-5*[S659,S662] double phosphorylation-site alterations in combination with *egfp*::*gpr-1*, a CRISPR/Cas9-mediated knockin allele of the endogenous *gpr-1* locus. Immunohistochemical detection of eGFP and LIN-5 showed normal colocalization of LIN-5 and GPR-1 in phosphomimetic *lin-5*[S659E,S662D] mutants at the centrosomes and cell cortex (Fig 5 and S7 Fig; note that LIN-5 becomes clearly visible at the cortex only after the one-cell stage). In contrast, in *lin-5*[S659A,S662A] mutant embryos, GPR-1 localized to the cortex but no longer accumulated at the centrosomes (Fig 5). Notably, GPR-1/2 localization at the cortex primarily depends on association with the GOA-1 and GPA-16 Gα proteins and is required for pulling forces, whereas ASPM-1–LIN-5 anchors GPR-1/2 at the centrosome without early embryonic requirement [21,37]. Thus, while the loss of centrosomal GPR-1 appears to confirm a reduced binding affinity for LIN-5[S659A,S662A] compared to wild type LIN-5, the reduced pulling forces likely result from a similarly reduced affinity between these proteins at the cortex. Nevertheless, LIN-5 still localized to the cortex in *lin-5*[S659A,S662A] mutants (S7 Fig). This likely reflects different dynamics of the two complexes; with rapid exchange of LIN-5 at the cortex while centrosomal GPR-1/2 accumulation likely depends on prolonged LIN-5 association. The combined observations in yeast two-hybrid assays, phenotypic analyses, and protein localization studies strongly indicate that phosphorylation of LIN-5 residues S659 and S662 contributes to the affinity of the LIN-5/GPR-1/2 interaction *in vivo*.

### A potential LIN-5 phosphorylation-dephosphorylation cycle in dynein recruitment

Characterization of the CDK-1 phosphorylation site mutants required a different strategy, as homozygous *lin-5*[T168A,T181A] and *lin-5*[T168D,T181D] mutants are fully sterile. To be able to examine the effects of these mutations in early embryos, we created trans-heterozygotes carrying these mutations and *egfp*::*lin-5*, a functional CRISPR/Cas9-generated knockin allele of endogenous *lin-5*. The *egfp*::*lin-5* allele served both as a visible balancer for the *lin-5* phosphorylation site mutations, and allowed selective knockdown of functional *lin-5* by RNAi against *egfp*. This strategy allowed us to obtain and characterize early embryos with CDK1-phosphorylation site alterations in LIN-5.

**Fig 6:**
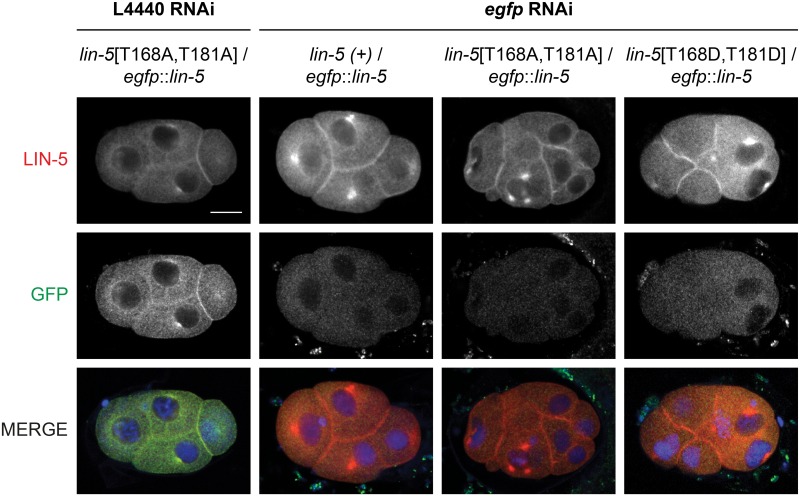
T168 and T181 mutants show normal subcellular LIN-5 localization. Immunohistochemical staining of embryos heterozygous for *egfp*::*lin-5* and wild type or phosphomutant *lin-5* as indicated. L4 animals were treated with feeding RNAi against control or *egfp* RNAi to specifically remove *egfp*::*lin-5* function, 48 hours before fixation of their embryos. Representative fluorescence microscopy images of embryos stained with anti-LIN-5 (red) and anti-GFP (green) antibodies, and DAPI to visualize DNA. All images taken with same exposure time, objective and magnification. Anterior to the left, scale bars 10 μm.

Control immunohistochemical staining experiments confirmed that *egfp* RNAi treatment of homozygous *egfp*::*lin-5* adults completely removed LIN-5 and eGFP from the offspring (S8 Fig). Following *egfp* RNAi treatment of heterozygous animals with wild type *lin-5 (lin-5(+) / egfp*::*lin-5*), LIN-5 localized normally, but the eGFP staining was lost (Fig 6). These results demonstrate that the RNAi effect remains specific for *egfp*::*lin-5* and does not carry over to the untagged *lin-5* allele. Testing balanced *lin-5*[T168A,T181A] and *lin-5*[T168D,T181D] animals the same way, we observed that the mutant LIN-5 proteins are expressed and localize as normal to the cortex and centrosomes, while the early embryonic divisions were clearly defective (Fig 6 and S5C Fig). Interestingly, *lin-5*[T168A,T181A] and *lin-5*[T168D,T181D] showed similar abnormalities, emphasizing the critical role for the *in vivo* phosphorylated threonine residues at these positions.

Using the above-described method, we also performed live imaging by time-lapse DIC microscopy and spindle severing experiments with *lin-5*[T168A,T181A] and *lin-5*[T168D,T181D] mutant embryos. Again, the defects observed in both mutants resembled *lin-5* strong loss-of-function [19,26], and cortical pulling forces were greatly reduced in both mutants (Fig 4E and S7 and S8 Videos). In contrast, homozygous *lin-5*[T168S,T181S] mutants showed normal spindle pulling forces (Fig 4E and S9 Video). This indicates that the two threonine residues are not essential per se, but phosphorylation and de-phosphorylation at these sites is likely critical. The *lin-5*[T168S,T181S] mutants did show dampened spindle oscillation, which might result from somewhat different kinetics of threonine versus serine phosphorylation and dephosphorylation in CDK1 substrates [38].

**Fig 7:**
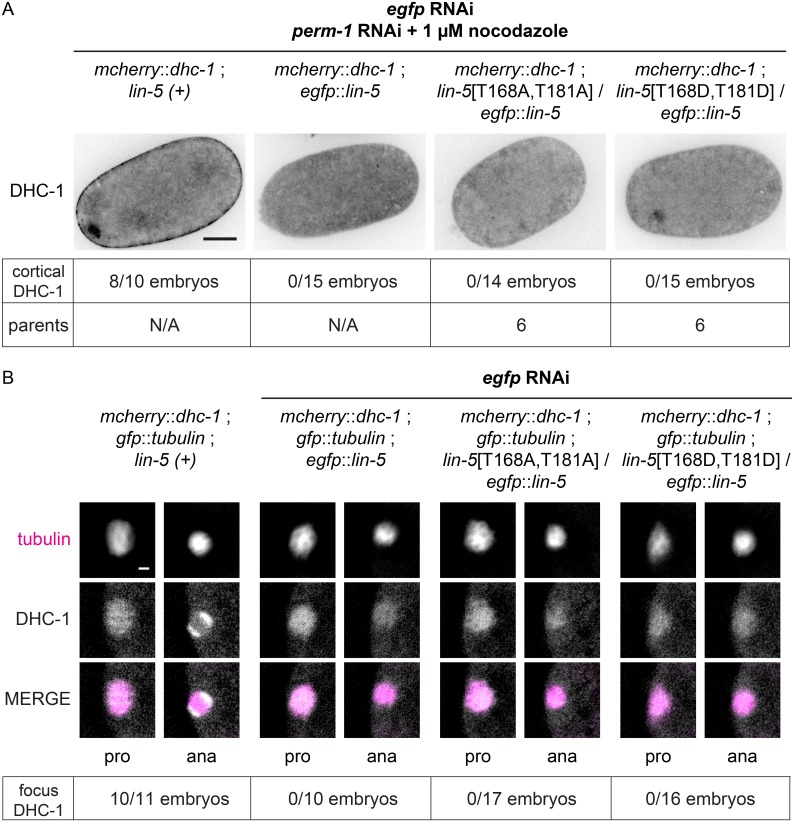
DHC-1 fails to accumulate at meiotic spindle poles in LIN-5 T168, T181 phosphosite mutants. **(A)** Representative still images of *mcherry*::*dhc-1; lin-5*[T168,T181] one-cell embryos treated with *perm-1* RNAi, *egfp* RNAi and 1 μM nocodazole, and imaged by spinning disk confocal microscopy. The number of embryos with cortical mCherry::DHC-1 is indicated, with “parents” indicating the number of animals from which embryos were analyzed. All images were taken with the same objective, magnification, and exposure time. Anterior to the left, scale bars 10 μm. **(B)** Representative snapshots of live imaging of *mcherry*::*dhc-1; tubulin*::*gfp; lin-5*[T168,T181] meiotic embryos treated with *egfp* RNAi, and imaged by wide field microscopy. Time interval between prophase and anaphase is 100 s. All images were taken with same exposure time, objective and magnification. Anterior to the left, scale bars 2 μm.

Because the N-terminus of LIN-5 is implicated in the recruitment of dynein [9], we crossed both mutants with an *mCherry*::*dhc-1* strain, in which the mCherry tag was introduced into the endogenous dynein heavy chain gene by CRISPR/Cas9-mediated knockin. This homozygous *mCherry*::*dhc-1* strain is viable and develops as normal. mCherry::DHC-1 was diffusely detected in the cytoplasm, and distinctly localized at the nuclear envelope, kinetochores, astral microtubules, spindle poles and cell cortex. Localization of dynein was dynamic during all stages of mitosis, but cortical dynein was barely detectable at the one-cell stage. However, following treatment of permeabilized embryos with nocodazole to depolymerize microtubules, mCherry::DHC-1 accumulated on the cell cortex of one-cell embryos in metaphase and anaphase (Fig 7A and S9 Fig). Strikingly, this cortical dynein localization was abolished by *lin-5* RNAi, and did not occur in *lin-5*[T168A,T181A] and *lin-5*[T168D,T181D] mutant embryos (Fig 7A). Since these mutant LIN-5 forms localize to the cell cortex, T168 and T181 are critical for the function of LIN-5 as a cortical dynein anchor.

In addition to cortical localization of dynein in mitosis, LIN-5 is also required for dynein recruitment to the poles of the meiotic spindle [37]. Accumulation of dynein at the spindle poles, as well as the cell cortex, occurs coincident with anaphase onset of meiosis I and II, and is needed for spindle rotation and expulsion of chromosomes into a polar body [37,39,40]. While homozygous *lin-5*[T168S,T181S] mutants showed normal meiosis, we observed polar body absence and abnormally large polar bodies in eGFP::LIN-5-depleted *lin-5*[T168A,T181A] and *lin-5*[T168D,T181A] embryos, consistent with *lin-5* loss of function. To examine meiotic spindle rotation and dynein localization in such embryos, we combined the *lin-5* mutations, balanced by *egfp*::*lin-5*, with homozygous *gfp*::*tbb-2* β—tubulin and mCherry::DHC-1 dynein reporters (Fig 7B and S10–S21 Videos). In a control strain with wild type LIN-5, spindle rotation and dynein accumulation occurred in 10 of 11 embryos (the one exception showed rotation but only weak mCherry::DHC-1 accumulation) (Fig 7B left and S10–S12 Videos). Examination of *egfp* RNAi treated *egfp*::*lin-5* embryos with combined DIC and fluorescence microscopy revealed normal diffuse association of DHC-1 with the meiotic spindle in meiotic prophase, followed by gradual loss of mCherry::DHC-1 from the anaphase spindle, rather than accumulation of dynein at the poles. The failure in dynein localization coincided with failure to rotate the meiotic spindle (Fig 7B). These results agree with our previously reported meiotic *lin-5* RNAi phenotype [21,37], although this time we also observed abnormally elongated meiotic spindles in meiosis II in a subset of the embryos, as has been reported for dynein complex subunits [40]. eGFP::LIN-5-depleted *lin-5*[T168A,T181A] and *lin-5*[T168D,T181D] embryos were indistinguishable from *lin-5* knockdown mutants (Fig 7B and S13–S21 Videos).

In conclusion, substitution of LIN-5 T168 and T181 with non-phosphorylatable alanine or phosphomimetic aspartic acid residues creates a severe defect in LIN-5-mediated dynein recruitment. In contrast, replacement of the same residues with phosphorylatable serine residues did not compromise LIN-5 function (Fig 4A, 4B and 4E). Combined with the available literature [37,39,41], these data point to CDK-1-mediated phosphorylation and subsequent dephosphorylation of the LIN-5 N-terminus as a critical step in dynein recruitment to the meiotic spindle and cell cortex (see below).

## Discussion

**Fig 8:**
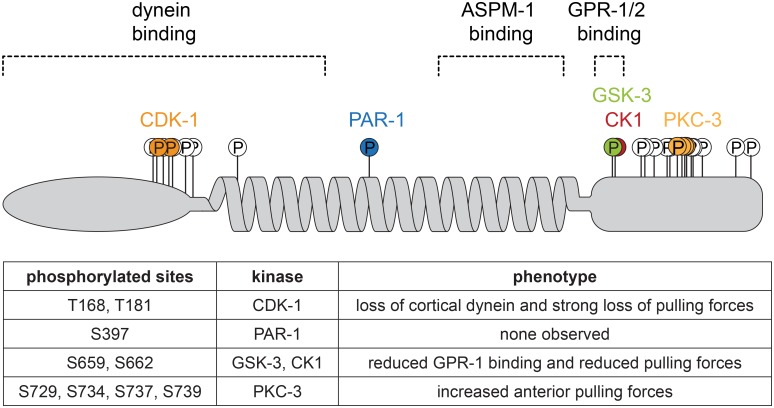
Summary of the proposed critical phosphorylations of LIN-5 by CDK-1, PAR-1, GSK3, CK1 and PKC-3. Overview of how phosphorylation-site mutations affect LIN-5 function *in vivo* in regulating cortical pulling forces that position the mitotic spindle and contribute to chromosome segregation.

In this study, we investigated whether the extensive *in vivo* phosphorylation of the LIN-5^NuMA^ protein is important for chromosome segregation and cell cleavage plane determination. We combined *in vivo* and *in vitro* phosphorylation analysis, identified critical phosphorylated LIN-5 residues by complementation, and defined the LIN-5–GPR-1 interaction domain by reverse yeast two-hybrid screening. Using this information, we created phosphosite mutants and tagged alleles by genetic engineering, and determined the *in vivo* contribution of individual phosphorylated residues by protein localization studies, time-lapse microscopy and spindle severing experiments. The combined data indicate that a variety of cell cycle and polarity kinases phosphorylate LIN-5, with specific phosphorylations promoting pulling force generation while others inhibit LIN-5 function. The combined phosphorylations of the LIN-5 N-terminus and C-terminus are critical in the spatiotemporal control of cortical pulling forces, and thereby for correct chromosome segregation and spindle positioning (Fig 8).

CRISPR/Cas9-mediated genomic engineering has added an important tool to a powerful genetic system, and more efficient procedures are continuously developed [31,42–46]. The use of CRISPR/Cas9 allowed us to precisely alter one or two codons of specific serine/threonine residues within the normal genetic background. Using knockin alleles eliminates unwanted effects of transgene overexpression or silencing. In particular transgene silencing has long hampered *lin-5* studies and was also observed in our complementation studies. Transgene expression levels that are close to a threshold level may explain why the *lin-5*[S659A,S662A] mutation showed a strong loss-of-function phenotype, while the effect of the same mutations introduced in the endogenous locus was less severe. In addition to phosphosite mutations, we also created tagged endogenous alleles of *lin-5*, *gpr-1* and *dhc-1* for fluorescent fusion protein expression. This allowed the development of a novel method for analysis of early lethal mutations. This method makes use of a functional eGFP-tagged allele, which acts as a visible balancer and allows the specific removal of wild type function by *egfp* RNAi.

In a previous study, we revealed *in vivo* kinase activity through differential labeling of *C*. *elegans* cultures with stable nitrogen isotopes, followed by kinase knockdown and quantitative analysis of phosphopeptides by mass spectrometry [15]. This strategy worked well for PKC-3, but various limitations can prevent detection of kinase-substrate relations *in vivo*. The phosphorylation of LIN-5 by PAR-1 was missed in our previous analysis, because of overlap between the relevant LIN-5 phosphopeptides and unrelated peptides. Identification of mitotic substrates of CDK-1 is difficult *in vivo*, because CDK-1 knockdown results in complete sterility and arrest of fertilized oocytes before completion of meiosis [47]. Casein kinase I, in turn, is represented by 87 family members in *C*. *elegans* [48], making it less likely that knockdown experiments will reveal a quantitative difference in substrate phosphorylation. The *in vitro* kinase assays in the current study revealed candidate kinases that were otherwise difficult to detect. The PAR-1 *in vitro* kinase assays pointed to a specific LIN-5 phosphorylation that was subsequently confirmed by our *in vivo* data. The *in vitro* phosphorylation of peptides with single phosphorylated residues was instrumental in detecting a probable two-step mechanism for S662 phosphorylation by CKI, following a priming phosphorylation by GSK3. Thus, while detecting direct phosphorylation *in vivo* remains the ultimate goal, *in vitro* assays continue to provide meaningful insight.

The combined *in vitro* and *in vivo* kinase analyses strongly suggest that PAR-1 phosphorylates LIN-5 at serine 397. Replacing this serine with non-phosphorylatable alanine or phosphomimetic glutamic acid apparently did not affect viability, development, cell division, chromosome segregation or spindle pulling forces. In fact, many phosphorylations that occur *in vivo* may be bystander rather than regulatory events, and determining which phosphorylations are critical *in vivo* has received great attention in the current study. The first selection came from alanine substitution mutagenesis combined with complementation of a *lin-5* null mutation. This revealed that 4 of the 25 phosphorylated residues are critical for LIN-5 function. As we could only score larval divisions in this assay, we cannot exclude that additional phosphorylations may be critical during embryogenesis. Remarkably, 2 of the 4 critical residues form part of a probable GPR-1/2 binding domain, while the other 2 appear to mediate contact with dynein at the cortex.

We defined the GPR-1/2 binding domain through screening for LIN-5 residues that are essential for GPR-1 interaction in yeast two-hybrid assays. The strong clustering of missense mutations in this screen combined with results from deletion analyses suggests a short linear GPR-interaction epitope. This is in full agreement with results from crystal structure studies of the related NuMA-LGN complex. The TPR repeats in the N-terminal half of LGN form helix-turn-helix repeats that together organize into a superhelical bundle [49,50]. The inner surface of this bundle forms a binding channel for an extended NuMA peptide of 28 amino acids [49]. Many electrostatic and hydrogen interactions between side chains of the NuMA peptide and TPR motifs together provide a high affinity binding site.

The TPR-repeat interaction site in LIN-5 resembles that of NuMA in size, position, and overall amino-acid composition. The exact residues are not well-conserved, however, probably because the many amino acids that contribute weak interactions provide a limited biological constraint for the conservation of individual amino acids. Notably, the core of the binding site contains EPEQLDDW in human NuMA and SPDSLPDF in LIN-5, sharing three identical and two similar residues as well as negative charge. The NuMA peptide contains four acidic residues (D, E), while two D residues and two phosphorylated serines are negatively charged in the LIN-5 peptide.

Phosphorylation offers the opportunity to regulate LIN-5–GPR-1/2 binding. In fact, dual GSK3 and CK1 phosphorylation of the LRP6 Wnt-co-receptor regulates the interaction of LRP6 with axin [24,51]. We did not obtain evidence to support developmentally regulated LIN-5–GPR-1 binding. Alanine substitution of S659 and S662 significantly reduced spindle pulling forces, but division of the zygote, EMS blastomere and seam cells continued to be asymmetric. The latter types of divisions depend on the Wnt-β —catenin asymmetry pathway, which in EMS positions the spindle redundantly with *mes-1/src-1* signaling [34]. Even the combined *lin-5*[S659A,S662A] mutation and *mes-1* knockdown did not interfere with A-P positioning of the spindle in EMS. Moreover, we could functionally replace serine 659 and 662 with glutamic and aspartic acid, suggesting that charge, rather than phosphoregulation, is critical for GPR-1/2 interaction.

A contribution of CDK-1 phosphorylation in LIN-5 regulation was expected. CDK1/cyclin B kinases are the master regulators of mitosis that phosphorylate hundreds of substrate proteins [22,52,53]. The LIN-5 N- and C-terminus and corresponding domains in NuMA contain multiple CDK1 consensus sites. CDK1/cyclin B has been shown to regulate *Xenopus* and human NuMA through phosphorylation of the C-terminus [18,54]. Specifically, phosphorylation at T2055 interferes with the cortical localization of NuMA, thereby inhibiting dynein recruitment until CDK1/cyclin B is inactivated at the metaphase/anaphase transition [18]. Our results indicate that this temporal regulation may also involve critical phosphorylation of the dynein-interacting N-terminus of NuMA by CDK1/cyclin B.

In *C*. *elegans*, dynein recruitment to the meiotic spindle and cell cortex, as well as mitotic pulling forces, depend on activation of the anaphase promoting complex/cyclosome (APC/C), and inactivation of CDK-1/cyclin B [37,39,41]. Thus, phosphorylation of specific mitotic substrates by CDK-1/cyclin B is likely to inhibit dynein recruitment and pulling force generation. A recent study identified the p150 dynactin subunit as a likely candidate for inhibition by CDK-1/cyclin B phosphorylation [40]. Our results point to the LIN-5 N-terminus as another critical target for CDK-1 regulation. Supporting this conclusion, T168, T181 are part of CDK consensus sites, are phosphorylated *in vivo*, and are efficiently phosphorylated by CDK1/cyclin B *in vitro*. Substitution of LIN-5 T168 and T181 with phosphomimetic glutamic acid or aspartic acid residues resulted in strong loss of LIN-5 function, supporting that CDK-1 phosphorylation normally inhibits LIN-5. More surprising, an indistinguishable phenotype was observed following T168 and T181 replacement with non-phosphorylatable alanine. This could indicate that the threonine residues are critical for LIN-5 folding, or that phosphorylation of these threonines in the N-terminus also contributes to dynein recruitment. In stark contrast to alanine substitution, replacement of the same residues with phosphorylatable serine had no detectable effect on pulling forces, meiotic and mitotic cell divisions, viability and fertility. While other explanations are possible, these data are consistent with a required sequential CDK-1 phosphorylation and dephosphorylation of LIN-5 T168 and T181. Therefore, we propose a two-step model, in which CDK-1/cyclin B induces the assembly of a LIN-5 pre-force generating complex in prometaphase. Subsequent removal of the phosphates, which follows CDK inactivation by the APC/C at anaphase onset, promotes interaction of this complex with dynein.

Many of the lessons learned from studies in worms and flies have subsequently been found to apply broadly to the animal kingdom. The initial discovery of LIN-5 requirement in spindle positioning in *C*. *elegans* [19] has contributed to identifying similar functions for NuMA in mammalian systems [55]. It will be intriguing to find out to what extent the phosphoregulation of pulling forces translates from *C*. *elegans* to mammalian systems, and specifically whether reversible CDK1 phosphorylation of the NuMA N-terminus controls dynein interaction and spindle positioning.

## Materials and Methods

### *C*. *elegans* strains

Strains were cultured on nematode growth medium plates, seeded with *Escherichia coli* OP50 as previously described [56]. Animals were maintained at 20°C, unless stated otherwise. All strains used in this study are found in S1 Table. Genome modifications in strains SV1568, SV1569, SV1586, SV1588, SV1589, SV1590, SV1600, SV1619, SV1621, SV1622, SV1695 and SV1901 were introduced by making use of CRISPR/Cas9 genome editing as described below.

### Post-embryonic analysis of LIN-5 phosphomutants

For functional analysis of wild type and phosphomutant LIN-5, 5 ng/μl *Plin-5*::*gfp*::*lin-5* DNA, together with 5 ng/μl *Psur-5*::*dsRed* and 25 ng/μl Lambda DNA (Fermentas), was injected into the gonad of SV918 young adults. *Psur-5*::*dsRed* positive F1 progeny were selected making use of a fluorescence stereo microscope (Leica, MZ16F). After this, *lin-5*(*e1348*) homozygous animals were selected based on absence of pharyngeal *Pmyo-2*::*gfp*, expressed from the *mIn1* balancer chromosome. Rescue analysis of *lin-5* null animals was performed by Differential Interference Contrast and fluorescence microscopy, using a Zeiss Axioplan microscope. Intestinal nuclei were counted only in animals expressing *Psur-5*::*dsRed* in all intestinal nuclei. Vulval development was assayed for all animals L4 and older.

For quantification of cell numbers in CRISPR/Cas9 knockin mutants, asynchronous populations of animals were fixed, DNA stained with propidium iodide and intestinal and ventral cord nuclei were counted using a Zeiss Axioplan fluorescence microscope. Cells were counted at late larval stages. For the ventral cord, all nuclei of the P2-to-P10 daughter cells and juvenile motor neurons in the region between these cells were counted.

### *In vitro* and *in vivo* kinase assays

For *in vitro* CDK1 kinase assays, immunoprecipitations were performed from mitotic lysates of HeLa cells using mouse monoclonal anti cyclin B1 (GNS1) or beads alone as negative control. Immunoprecipitations were incubated for 30 min at 30°C with either Histone H1, bacterially produced GST or GST–LIN-5 in kinase buffer containing 50 mM HEPES at pH 7.5, 5 mM MgCl_2_, 2.5 mM MnCl_2_, 1 mM dithiothreitol, 50 μM ATP and 2.5 μCi [γ-^32^P] ATP. Reactions were terminated by the addition of SDS (5x sample buffer). For mass spectrometry analysis, no [γ-^32^P] ATP was added to the kinase assays and incubation time was prolonged to 2 hours at 30°C.

For *in vitro* GSK3 and CK1 kinase assays, peptides (RRRIRCGSPDSLPDFLADN) containing either unphosphorylated, phosphorylated S659 or phosphorylated S662 were used. Kinases were incubated for 30 min at 25°C with synthetic peptide in kinase buffer containing 200 μM ATP, 50 mM HEPES at pH 7.5, 10 mM MgCl_2_, 1 mM EGTA, 2 mM dithiothreitol, supplemented with 20 μCi [γ-^32^P] ATP for radioactive kinase assays. Reactions were terminated by the addition of SDS (4*x* sample buffer).

All other *in vitro* kinase assays were performed as previously described [15].

In short, kinases were incubated for 30 min at 25°C with bacterially produced GST or GST–LIN-5 in kinase buffer containing 200 μM ATP, 50 mM HEPES at pH 7.5, 10 mM MgCl_2_, 1 mM EGTA, 2 mM dithiothreitol, supplemented with 20 μCi [γ-^32^P] ATP for radioactive kinase assays. Reactions were terminated by the addition of SDS (4*x* sample buffer). For mass spectrometry analysis, no [γ-^32^P] ATP was added to the kinase assays and incubation time was prolonged to 2 hours at 25°C. Kinases used in this study were: recombinant *C*. *elegans* PAR-1 (a kind gift from Erik Griffin and Geraldine Seydoux), and mammalian Aurora B (a kind gift from Susanne Lens), CK1 (New England Biolabs), CK2 (New England Biolabs), and GSK3 (New England Biolabs).

### In-gel digestion and phosphopeptide enrichment

Gel bands were cut and processed for protein in-gel digestion as described elsewhere [15]. Briefly, proteins were reduced with dithiothreitol and then alkylated with iodoacetamide. Trypsin was added at a concentration of 10 ng/μl and the mixture was digested overnight at 37°C. Subsequently, peptides were collected from the supernatants and a second extraction using 10% formic acid was performed. Phosphopeptides from LIN-5 were enriched using TiO_2_ chromatography [57]. Basically, home-made GELoader tips (Eppendorf, Hamburg, Germany) were packed with TiO2 beads (5 μm, INERTSIL). Peptides were loaded in 10% formic acid and subsequently washed with 20 μl of 80% acetonitrile, 0.1% trifluoroacetic acid (Fluka, Sigma-Aldrich). Phosphopeptides were then eluted twice with 20 μl of 1.25% ammonia solution (Merck, Germany), pH 10.5, and 3 μl of 100% formic acid was finally added to acidify the samples.

### Mass-spectrometry analysis

Nanoflow LC-MS/MS was carried out by coupling an Agilent 1100 HPLC system (Agilent Technologies, Waldbronn, Germany) to an LTQ-Orbitrap XL mass spectrometer (Thermo Electron, Bremen, Germany). Peptide samples were delivered to a trap column (AquaTM C18, 5 μm (Phenomenex, Torrance, CA); 20 mm x 100-μm inner diameter, packed in house) at 5 μl/min in 100% solvent A (0.1 M acetic acid in water). Next, peptides eluted from the trap column onto an analytical column (ReproSil-Pur C18-AQ, 3μm (Dr. Maisch GmbH, Ammerbuch, Germany); 40 cm x 50-μm inner diameter, packed in house) at ~100 nl/min in a 90 min or 3 h gradient from 0 to 40% solvent B (0.1 M acetic acid in 8:2 (v/v) acetonitrile/water). The eluent was sprayed via distal coated emitter tips butt-connected to the analytical column. The mass-spectrometer was operated in data-dependent mode, automatically switching between MS and MS/MS. Full-scan MS spectra (from m/z 300 to 1500) were acquired in the Orbitrap with a resolution of 60,000 at m/z 400 after accumulation to target value of 500,000 in the linear ion trap. The five most intense ions at a threshold above 5000 were selected for collision-induced fragmentation in the linear ion trap at a normalized collision energy of 35% after accumulation to a target value of 10,000. Peak lists were created from raw files with MaxQuant^43^. Peptide identification was carried out with Mascot (Matrix Science) against a *Caenorhabditis elegans* protein database (http://www.wormbase.org) supplemented with all the frequently observed contaminants in MS (23,502 protein sequences in total). The following parameters were used: 10 ppm precursor mass tolerance, 0.6 Da fragment ion tolerance, up to 3 missed cleavages, carbamidomethyl cysteine as fixed modification, oxidized methionine, phosphorylated serine, threonine and tyrosine as variable modifications. Alternatively, MaxQuant and its search engine Andromeda was also employed for peptide identification and quantification. Data are available via ProteomeXchange with identifier PXD004906

### Yeast two-hybrid fragment analysis

Full length *gpr-1* was PCR amplified from the ORFeome library (kind gift from Marc Vidal) using KOD polymerase (Novagen) and cloned into bait vector pPC97. Fragments of *lin-5* were PCR amplified from the ORFeome library with KOD polymerase (Novagen) and cloned into prey vector pPC86-AN [58]. DB::GPR-1- and AD::LIN-5-encoding plasmids were transformed sequentially into yeast strain Y8930 [58]. Positive interactions were identified on the basis of the activation of the HIS3 and ADE2 reporter genes, indicated by growth on synthetic complete –leucine −tryptophan −histidine + 2 mM 3-Amino-1,2,4-triazole (Sc −leu−trp−his + 3-AT) and synthetic complete –leucine −tryptophan −adenine (Sc −leu−trp−ade) plates.

### Generation and verification of yeast two-hybrid *lin-5* mutants

To generate mutant clones, PCR was performed on pVP054 (pPC86-AN containing nucleotides 1821–2466 encoding amino acid 609–821 of *lin-5*) with increased MgCl_2_ concentration of 7 mM. PCR products were cloned into pPC86-AN and transformed to DH5α competent cells. Bacterial colonies were collected and the DNA was isolated using a Nucleobond Xtra DNA purification kit (Macherey-Nagel). Bacterial clones were transformed into MB004 (Y8930 in which ADE2 is replaced by URA3 by homologous recombination) containing DB::GPR-1 and plated on synthetic complete –leucine −tryptophan + 2 g/l 5-fluoroacetic acid (Sc −leu−trp + FOA). Colonies were picked and spotted to synthetic complete –leucine −tryptophan (Sc −leu−trp) plates for validation, PCR and sequencing. Clones with single amino acid changes were re-tested by PCR amplification of the fragment and re-cloning into pPC86-AN. Interaction deficient alleles were identified on the basis of no activation of the HIS3 or URA3 reporter gene, indicated by absence of growth on Sc −leu−trp−his + 3-AT plates and synthetic complete –leucine −tryptophan –uracil (Sc −leu−trp−ura) plates.

### Validation of yeast two-hybrid full length mutants

For generation of full length mutant clones, plasmids containing fragment clones with selected point mutations were digested and cloned into pVP055 (pPC86-AN containing nucleotides 1–2466 encoding amino acid 1–821 of *lin-5*). Phosphomutants were generated by either site-directed mutagenesis of pVP055 or Gibson assembly into pVP055 of short regions of *lin-5* with point mutations carried in the overlapping region. DB::GPR-1 and AD::LIN-5-encoding plasmids were transformed sequentially into yeast strain MB004. Interaction deficient alleles were identified on the basis of no activation of the HIS3 or URA3 reporter gene, indicated by absence of growth on Sc −leu−trp−his + 3-AT plates and Sc −leu−trp−ura plates.

### Generation of CRISPR/Cas9 repair templates

CRISPR repair constructs were inserted into the pBSK vector using Gibson Assembly (New England Biolabs). Homologous arms of at least 1500 bp upstream and downstream of the CRISPR/Cas9 cleavage site were amplified from either cosmid C03G3 (for *lin-5* constructs) or *C*. *elegans* genomic DNA using KOD Polymerase (Novagen). Linkers containing the altered cleavage sites and point mutations were synthesized (Integrated DNA technologies). For *fkbp*::*egfp*::*gpr-1*, codon-optimized *fkbp* was synthesized (Integrated DNA technologies) and codon-optimized *egfp* was amplified from pMA-*egfp* (a kind gift from Anthony Hyman). For *egfp*::*lin-5*, codon-optimized *egfp* was amplified from pMA-*egfp*. For *mcherry*::*dhc-1*, codon-optimized *mcherry* was amplified from TH0563-PAZ-*mCherry* (a kind gift from Anthony Hyman). Mismatches were introduced in the sgRNA target site to prevent cleavage of knockin alleles. All plasmids and primers used for cloning are available upon request.

### CRISPR/Cas9 genome editing

Young adults were injected with a solution containing the following injection mix: 30–50 ng/μl *Peft-3*::Cas9 (Addgene 46168; [59], 30–100 ng/μl u6::sgRNA with appropriate target for *dhc-1*, *gpr-1* and *lin-5*, 30–50 ng/μl repair template and 2.5 ng/μl *pmyo-2*::*tdTomato*. Progeny of animals that express tdTomato were picked to new plates 3–4 days post injection. PCRs with primers diagnostic for homologous recombination at the endogenous locus were performed on F2-F3 populations, where one primer targeted the altered basepairs in the sgRNA site, point mutation or fluorescent tag and the other targeted a region just outside the homology arm. All primers used for genome editing are available upon request.

### Preparation of protein lysates for western blot analysis

For yeast protein lysates, cultures were grown overnight at 30°C. Yeast cells corresponding to 4 OD of culture were harvested, treated with Sodium hydroxide and resuspended in 100 2X sample buffer containing β-mercaptoethanol [60].

For *C*. *elegans* protein lysates, strains SV1569, SV1663 and SV1664 were grown at 20°C one generation on NGM plates seeded with HB101, followed by a second generation in S-Medium with HB101 bacteria. Gravid adults were harvested and embryos were isolated by hypochlorite treatment. Embryo pellets were snap frozen in liquid nitrogen, grinded using mortar and pestle and resuspended in 5 ml fresh lysis buffer (containing 20 mM Tris-HCl pH 7.8, 250 mM NaCl, 15% glycerol, 0.5% IGEPAL, 0.5 mM EDTA, 50 mM Sodium fluoride, 1 mM β-mercaptoethanol and protease inhibitors (Roche complete, Mini, EDTA-free)). The suspension was passed through a French press 3 times, and the lysate was cleared at 13,000 rpm for 15 min at 4°C.

### Western blot analysis

Protein samples were separated on gradient acrylamide gels and subjected to western blotting on polyvinylidene difluoride membrane (Immobilon-P, Millipore). Membranes were blocked with 5% skim milk in PBST for 1 hour at room temperature, or overnight at 4°C for stripped blots. For protein detection, primary antibodies used in this study were: mouse anti–LIN-5 (1:1000) [19] and rabbit anti-Tubulin (1:1000, Abcam) for stripped blots. Secondary antibodies used were: donkey anti-mouse HRP (1:5000, Abcam) and goat anti-rabbit HRP (1:5000, Jackson Immunoresearch). Proteins were dectected with Signalfire Plus chemiluminescent detection (Cell Signaling Technologies) and a Chemidoc MP Imager (Bio-Rad).

### Time-lapse imaging and live-cell imaging

DIC time-lapse imaging was performed on strains N2, SV1568, SV1588, SV1590 and SV1600. Animals were grown overnight at 25°C. RNAi feeding of N2 against *gsk-3* was performed with the bacterial clone from the Orfeome-based RNAi Library [61,62]. L4 animals were grown for approximately 32 hours at 15°C before shifting overnight to 25°C for imaging, except SV1901 which was kept at 20°C. Embryos were dissected from adults in a solution of 0.8x egg salt (containing 94 mM NaCl, 32 mM KCl, 2.7 mM CaCl_2_, 2.7 mM MgCl_2_, 4 mM HEPES, pH 7.5; [63]) on coverslips and mounted on slides with 3% agarose prepared with egg salt. Embryos were imaged with 5s time intervals with a 100x/1.4 NA lens on a Zeiss microscope at 20°C. Relative positions of the spindle and furrow were analyzed manually using ImageJ.

Live-cell imaging of EMS rotation was performed on strains SV1783, SV1784 and SV1785. L4 animals were grown overnight at 20°C. Embryos were dissected from young adults as above and imaged with a 100x/1.4 NA lens on a Zeiss microscope at 20°C. Spindle rotation in EMS was followed over time with images taken at several time points.

Live-cell imaging of microtubule depolymerization upon nocodazole treatment was performed on strain AZ244. Young adult animals were injected with dsRNA [64] against *perm-1* and grown for 20 hours at 15°C. Embryos were dissected from young adults in a solution of 0.8x egg salt containing 1 μM nocodazole on coverslips and mounted on concave slides. Embryos were imaged with a 60x/1.4 NA lens on a Nikon Eclipse Ti microscope with Perfect Focus System and Yokogawa CSU-X1-A1 spinning disk confocal head at room temperature.

Live-cell imaging of mitotic DHC-1 localization was performed on strains SV1619, SV1635, SV1638 and SV1639. Young adult animals were injected with dsRNA [64] against *perm-1* and *egfp* and grown for 20 hours at 15°C. Embryos were dissected from young adults in a solution of 0.8x egg salt containing 1 μM nocodazole on coverslips and mounted on concave slides. Still images of mitotic embryos in metaphase were taken within minutes after nocodazole addition. Eliminating nonspecific toxic effects, embryos on the same slide at the same time continued nuclear envelope degradation, and *perm-1(RNAi)* embryos continued embryonic development in the absence of nocodazole.

Embryos were imaged with a 60x/1.4 NA lens on a Nikon Eclipse Ti microscope with Perfect Focus System and Yokogawa CSU-X1-A1 spinning disk confocal head at room temperature. mCherry::DHC-1 localization was analyzed in mitotic one-cell embryos after nuclear envelope breakdown. Mitotic embryos were also identified based on presence of a polar body, enlarged centrosomes and remnant of the mitotic spindle.

Live-cell imaging of meiotic DHC-1 localization was performed on strains SV1702, SV1898, SV1899 and SV1902. For RNAi treated animals, young adult animals were injected with dsRNA [64] against *egfp* and grown for 24 hours at 15°C. Embryos were dissected from young adults as above and imaged with 10s time intervals with a 100x/1.4 NA lens on a Zeiss microscope at 20°C. For images presented in Fig 7B and S12, S15, S18 and S21 Videos, images were processed by subtracting a Gaussian-blur filtered image (Sigma(Radius): 20) using ImageJ. S10, S11, S13, S14, S16, S17, S19 and S20 Videos represent the unprocessed files.

### UV laser spindle ablation

Spindle severing with a UV laser microbeam was performed on strains SV1585, SV1594, SV1596, SV1618, SV1700 and SV1701 essentially as previously described [13]. RNAi feeding of SV1700 and SV1701 against *egfp* was performed with a bacterial clone containing full length *egfp* in the L4440 double T7 plasmid [61]. L4 animals were grown on RNAi for approximately 48 hours. For analysis of spindle pulling forces, animals were kept at 25°C for 24 h before ablations. Spindle ablations were carried out at 25°C (Fig 4D) or 20°C (Fig 4E) on a spinning disk confocal microscope. The spindle midzones were severed at anaphase onset and images of GFP-β-tubulin were taken at 0.5 s intervals. For analysis, the position of the spindle poles was automatically tracked using the MTrack2 plugin in ImageJ. Peak velocities of the anterior and posterior spindle poles were determined within a 12.5 s time frame after ablation. Representative videos for every strains are shown in S2–S9 Videos.

Microscope setup: Nikon Eclipse Ti microscope with Perfect Focus System, Yokogawa CSU-X1-A1 spinning disk confocal head, S Fluor 100x N.A. 0.5–1.3 objective (at 1.3), Photometrics Evolve 512 EMCCD camera, Cobolt Calypso 491 nm (100 mW) and Teem Photonics 355 nm Q-switched pulsed lasers, ILas system (Roper Scientific France/ PICT-IBiSA, Institut Curie) to control the UV laser, ET-GFP (49002) filter, ASI motorized stage MS-2000-XYZ with Piezo Top Plate with Tokai Hit INUBG2E-ZILCS Stage Top Incubator (controlled at 25°C), controlled by MetaMorph 7.7 software.

### Antibodies and immunohistochemistry

For immunostaining, embryos were dissected from adults in 8 μl of water on poly-L-lysine–coated slides. Embryos were freeze-cracked and fixed for 5 min in methanol at −20°C and then for 20 min in acetone at −20°C. After fixation, embryos were rehydrated in phosphate-buffered saline (PBS) containing 0.05% Tween-20 (PBST) and blocked with blocking solution (PBST containing 1% bovine serum albumin and 1% goat serum [Sigma-Aldrich]) for 1 h. Embryos were stained with primary and secondary antibodies for 1 h and washed after each incubation with PBST four times, 15 min each time. Finally, the embryos were embedded in ProLong Gold Antifade containing 4′,6-diamidino-2-phenylindole (DAPI). Primary antibodies used in this study were: mouse anti–LIN-5 (1:10; [19] and rabbit anti-GFP (1:500, Life Technologies). Secondary antibodies were used at a concentration of 1:500. Secondary antibodies used were: goat anti-rabbit Alexa Fluor 488, and goat anti-mouse Alexa Fluor 568 (Invitrogen). Images were taken with a 63x/1.4 NA lens on a Zeiss confocal microscope.

### Immunohistochemistry quantification of LIN-5 levels

Embryos were dissected and stained with antibodies as described above. Images were taken with a 63x/1.4 NA lens on a Zeiss confocal microscope using identical microscope setting for all images taken for every secondary antibody. Mean intensity of LIN-5 was measured using ImageJ by selecting fixed size regions that depended on the developmental stage. For every embryo 2 centrosomes and cytoplasmic regions were quantified.

## Supporting Information

S1 Fig Phosphorylation of LIN-5 controls post-embryonic divisions in the vulva. Quantification of vulval development in heterozygous *lin-5*(*e1348*) / *mIn1* (Wild type), homozygous *lin-5*(*e1348*) animals, and homozygous *lin-5*(*e1348*) animals expressing *gfp*::*lin-5* transgenes.
(TIF)

S2 Fig CDK1 phosphorylates LIN-5 *in vitro*. **(A)** *In vitro* CDK1 kinase assay with recombinant GST-LIN-5, GST alone, or Histone H1. Left; coomassie brilliant blue (CBB)-stained gel. Right; autoradiogram. **(B)** List of LIN-5 phosphopeptides identified by mass spectrometry analysis of CDK1 *in vitro* kinase assay with GST-LIN-5. Phosphopeptides are shown with individual Mascot Scores. Only Mascot Scores above 20 were accepted as reliable peptide identifications.
(TIF)

S3 Fig PAR-1 phosphorylates LIN-5 *in vivo*. Log_2_ ratios for all of the quantified ^15^N/^14^N peptide pairs as a function of their mass-spectrometry intensities in the three LIN-5 immunoprecipitates. LIN-5 phosphopeptides are represented in red, and LIN-5 regular peptides are represented in blue. Peptides belonging to other proteins are shown in grey. Peptide intensities were calculated using the average of the ^14^N and ^15^N extracted ion chromatograms.
(TIF)

S4 Fig Summary of GPR-1 interaction-deficient LIN-5 alleles. **(A)** Summary of sequencing results of all interaction-deficient alleles of LIN-5 identified in the reverse yeast two-hybrid assay before further validation. **(B)** Overview of all interaction deficient alleles of LIN-5 containing a single amino acid change identified in the reverse yeast two-hybrid assay before further validation. Validation is shown in Fig 3B.
(TIF)

S5 Fig Detection of protein levels in LIN-5 mutants. **(A)** Western blots of lysates of yeast clones containing the indicated LIN-5 expression constructs, probed for LIN-5 and tubulin (loading control) levels. **(B)** Western blots of *C*. *elegans* lysates with detection of LIN-5 and tubulin (loading control). **(C)** Quantification of mean intensity of immunostainings of *lin-5*[mutant T168,T181] / *egfp*::*lin-5* embryos treated with *egfp* RNAi and stained with LIN-5 antibodies. Graphs indicate single values for centrosomes and cytoplasm in one- and two-cell embryos.
(TIF)

S6 Fig Phenotypical analysis shows developmental defects in LIN-5 phosphomutants. **(A)** Averages of quantification of intestinal nuclei and P-cells plus juvenile motor neurons (P2-P10 region) by propidium iodide staining in wild type, homozygous LIN-5 phosphorylation mutants, and homozygous *lin-5*(*e1348*) null animals. Statistical analysis in s.e.m., analyzed by Graphpad PRISM. **(B)** Statistical averages of DIC microscopy imaging of hallmarks of the first 2 embryonic divisions in LIN-5 phosphorylation mutants. Oscillations are plotted in percentage of embryo height, elongation and AB size as a percentage of embryo width, flattening and rotation as a total fraction of analyzed embryos. Statistical analysis in s.e.m., analyzed by Graphpad PRISM. **(C)** Quantification of EMS spindle rotation in *mes-1*(*bn74 ts*); *lin-5[S659*,*S662]* phosphorylation mutant and *mes-1*(*bn74 ts*); *gsk-3(RNAi)* embryos. Spindle rotation was quantified by live-imaging of the *gfp*::*tubulin* marker. Wt indicates number of embryos with wild type rotation, defective A-P indicates number of embryos with a failure to fully align in the anterior-posterior direction, defective L-R indicates number of embryos with a failure to rotate in the left-right direction.
(TIF)

S7 Fig Localization of LIN-5 and GPR-1 in phosphorylation mutants. Immunohistochemical staining of embryos expressing wild type or phosphomutant *lin-5* and endogenously tagged *egfp*::*gpr-1*. Representative images of four-cell embryos, stained with anti-LIN-5 (red) and anti-eGFP (green) antibodies, and DAPI to visualize DNA. All images taken with same exposure time, objective and magnification. Anterior to the left, ventral up.
(TIF)

S8 Fig Specific knockdown of eGFP::LIN-5 upon *egfp* RNAi. Immunohistochemical staining of heterozygous and homozygous *egfp*::*lin-5 C*. *elegans* embryos with anti-LIN-5 (red) and anti-GFP (green) antibodies, DNA stained with DAPI. Two representative embryos are shown for every condition. All images same objective and magnification, anterior to the left, scale bars 10 μm.
(TIF)

S9 Fig Cortical localization of mCherry::DHC-1 after nocodazole treatment. **(A)** Representative snapshots of live imaging of GFP::tubulin in *gfp*::*tubulin* one-cell embryos treated with or without *perm-1* RNAi + 1 μM nocodazole, and imaged by spinning disk confocal microscopy. Scale bars, 10 μm, all images with same objective and magnification. **(B)** Representative snapshots of live imaging of mCherry::DHC-1 in *mcherry*::*dhc-1*; *egfp*::*lin-5* one-cell embryos in prophase and metaphase treated with or without *perm-1* RNAi in the presence or absence of 1 μM nocodazole, and imaged by spinning disk confocal microscopy. All images taken with same objective and magnification, anterior to the left, scale bars 10 μm.
(TIF)

S1 Video Time-lapse imaging of developmental hallmarks in embryos. Video of DIC time-lapse microscopy imaging of hallmarks of the first 2 embryonic divisions in a wild type embryo as quantified in Fig 4C and S6 Fig. Time intervals between frames 5 s, frame rate video 7 fps. Anterior to the left.
(MP4)

S2 Video Spindle severing of control embryos. Representative video of UV laser spindle ablation experiments in a *lin-5*; *gfp*::*tubulin* embryo as quantified in Fig 4D. Time intervals between frames 500 ms, frame rate video 5 fps. Anterior to the left.
(AVI)

S3 Video Spindle severing of *lin-5*[S397A] embryos. Representative video of UV laser spindle ablation experiments in a *lin-5*[S397A]; *gfp*::*tubulin* embryo as quantified in Fig 4D. Time intervals between frames 500 ms, frame rate video 5 fps. Anterior to the left.
(AVI)

S4 Video Spindle severing of *lin-5*[S397E] embryos. Representative video of UV laser spindle ablation experiments in a *lin-5*[S397E]; *gfp*::*tubulin* embryo as quantified in Fig 4D. Time intervals between frames 500 ms, frame rate video 5 fps. Anterior to the left.
(AVI)

S5 Video Spindle severing of *lin-5*[S659A,S662A] embryos. Representative video of UV laser spindle ablation experiments in a *lin-5*[S659A,S662A]; *gfp*::*tubulin* embryo as quantified in Fig 4D. Time intervals between frames 500 ms, frame rate video 5 fps. Anterior to the left.
(AVI)

S6 Video Spindle severing of *lin-5*[S659E,S662D] embryos. Representative video of UV laser spindle ablation experiments in a *lin-5*[S659E,S662D]; *gfp*::*tubulin* embryo as quantified in Fig 4D. Time intervals between frames 500 ms, frame rate video 5 fps. Anterior to the left.
(AVI)

S7 Video Spindle severing of *lin-5*[T168A,T181A] embryos. Representative video of UV laser spindle ablation experiments in a *lin-5*[T168A,T181A]; *gfp*::*tubulin* embryo as quantified in Fig 4E. Time intervals between frames 500 ms, frame rate video 5 fps. Anterior to the left.
(AVI)

S8 Video Spindle severing of *lin-5*[T168D,T181D] embryos. Representative video of UV laser spindle ablation experiments in a *lin-5*[T168D,T181D]; *gfp*::*tubulin* embryo as quantified in Fig 4E. Time intervals between frames 500 ms, frame rate video 5 fps. Anterior to the left.
(AVI)

S9 Video Spindle severing of *lin-5*[T168S,T181S] embryos. Representative video of UV laser spindle ablation experiments in a *lin-5*[T168S,T181S]; *gfp*::*tubulin* embryo as quantified in Fig 4E. Time intervals between frames 500 ms, frame rate video 5 fps. Anterior to the left.
(AVI)

S10 Video Meiotic spindle localization of tubulin in wild type embryos. Representative video of GFP::tubulin in a *mcherry*::*dhc-1; gfp*::*tubulin* embryo as analyzed in Fig 7B. Time intervals between frames 10 s, frame rate video 4 fps. Anterior to the left.
(AVI)

S11 Video Meiotic spindle localization of dynein in wild type embryos. Representative video of mCherry::DHC-1 in a *mcherry*::*dhc-1; gfp*::*tubulin* embryo as analyzed in Fig 7B. Time intervals between frames 10 s, frame rate video 4 fps. Anterior to the left.
(AVI)

S12 Video Merges of meiotic spindle localization of tubulin and dynein in wild type embryos. Representative video of GFP::tubulin (magenta) and mCherry::DHC-1 (gray) in a *mcherry*::*dhc-1; gfp*::*tubulin* embryo as analyzed in Fig 7B. Time intervals between frames 10 s, frame rate video 4 fps. Anterior to the left.
(AVI)

S13 Video Meiotic spindle localization of tubulin in *lin-5* depleted embryos. Representative video of GFP::tubulin in a *mcherry*::*dhc-1; gfp*::*tubulin; egfp*::*lin-5* embryo treated with *egfp* RNAi as analyzed in Fig 7B. Time intervals between frames 10 s, frame rate video 4 fps. Anterior to the left.
(AVI)

S14 Video Meiotic spindle localization of dynein in *lin-5* depleted embryos. Representative video of mCherry DHC-1 in a *mcherry*::*dhc-1; gfp*::*tubulin; egfp*::*lin-5* embryo treated with *egfp* RNAi as analyzed in Fig 7B. Time intervals between frames 10 s, frame rate video 4 fps. Anterior to the left.
(AVI)

S15 Video Merges of meiotic spindle localization of tubulin and dynein in *lin-5* depleted embryos. Representative video of GFP::tubulin (magenta) and mCherry::DHC-1 (gray) in a *mcherry*::*dhc-1; gfp*::*tubulin; egfp*::*lin-5* embryo treated with *egfp* RNAi as analyzed in Fig 7B. Time intervals between frames 10 s, frame rate video 4 fps. Anterior to the left.
(AVI)

S16 Video Meiotic spindle localization of tubulin in *lin-5*[T168A,T181A] embryos. Representative video of GFP::tubulin in a *mcherry*::*dhc-1; gfp*::*tubulin; lin-5*[T168A,T181A] */ egfp*::*lin-5* embryo treated with *egfp* RNAi as analyzed in Fig 7B. Time intervals between frames 10 s, frame rate video 4 fps. Anterior to the left.
(AVI)

S17 Video Meiotic spindle localization of dynein in *lin-5*[T168A,T181A] embryos. Representative video of mCherry::DHC-1 in a *mcherry*::*dhc-1; gfp*::*tubulin; lin-5*[T168A,T181A] */ egfp*::*lin-5* embryo treated with *egfp* RNAi as analyzed in Fig 7B. Time intervals between frames 10 s, frame rate video 4 fps. Anterior to the left.
(AVI)

S18 Video Merges of meiotic spindle localization of tubulin and dynein in *lin-5*[T168A,T181A] embryos. Representative video of GFP::tubulin (magenta) and mCherry::DHC-1 (gray) in a *mcherry*::*dhc-1; gfp*::*tubulin; lin-5*[T168A,T181A] */ egfp*::*lin-5* embryo treated with *egfp* RNAi as analyzed in Fig 7B. Time intervals between frames 10 s, frame rate video 4 fps. Anterior to the left.
(AVI)

S19 Video Meiotic spindle localization of tubulin in *lin-5*[T168D,T181D] embryos. Representative video of GFP::tubulin in a *mcherry*::*dhc-1; gfp*::*tubulin; lin-5*[T168D,T181D] */ egfp*::*lin-5* embryo treated with *egfp* RNAi as analyzed in Fig 7B. Time intervals between frames 10 s, frame rate video 4 fps. Anterior to the left.
(AVI)

S20 Video Meiotic spindle localization of dynein in *lin-5*[T168D,T181D] embryos. Representative video of mCherry::DHC-1 in a *mcherry*::*dhc-1; gfp*::*tubulin; lin-5*[T168D,T181D] */ egfp*::*lin-5* embryo treated with *egfp* RNAi as analyzed in Fig 7B. Time intervals between frames 10 s, frame rate video 4 fps. Anterior to the left.
(AVI)

S21 Video Merges of meiotic spindle localization of tubulin and dynein in *lin-5*[T168D,T181D] embryos. Representative video of GFP::tubulin (magenta) and mCherry::DHC-1 (gray) in a *mcherry*::*dhc-1; gfp*::*tubulin; lin-5*[T168D,T181D] */ egfp*::*lin-5* embryo treated with *egfp* RNAi as analyzed in Fig 7B. Time intervals between frames 10 s, frame rate video 4 fps. Anterior to the left.
(AVI)

S1 Table Overview of *C*. *elegans* strains used in this study. Overview of all strains used in this study, including the corresponding figure. CRISPR/Cas9 genome engineered strains are indicated in the comment section.
(PDF)
