# Supplementary figures and images for: Multisite Phosphorylation of NuMA-Related LIN-5 Controls Mitotic Spindle Positioning in C. elegans
Source: PLoS Genet. 2016 Oct 6;12(10):e1006291. doi: 10.1371/journal.pgen.1006291 (PMC5053539; doi:10.1371/journal.pgen.1006291)

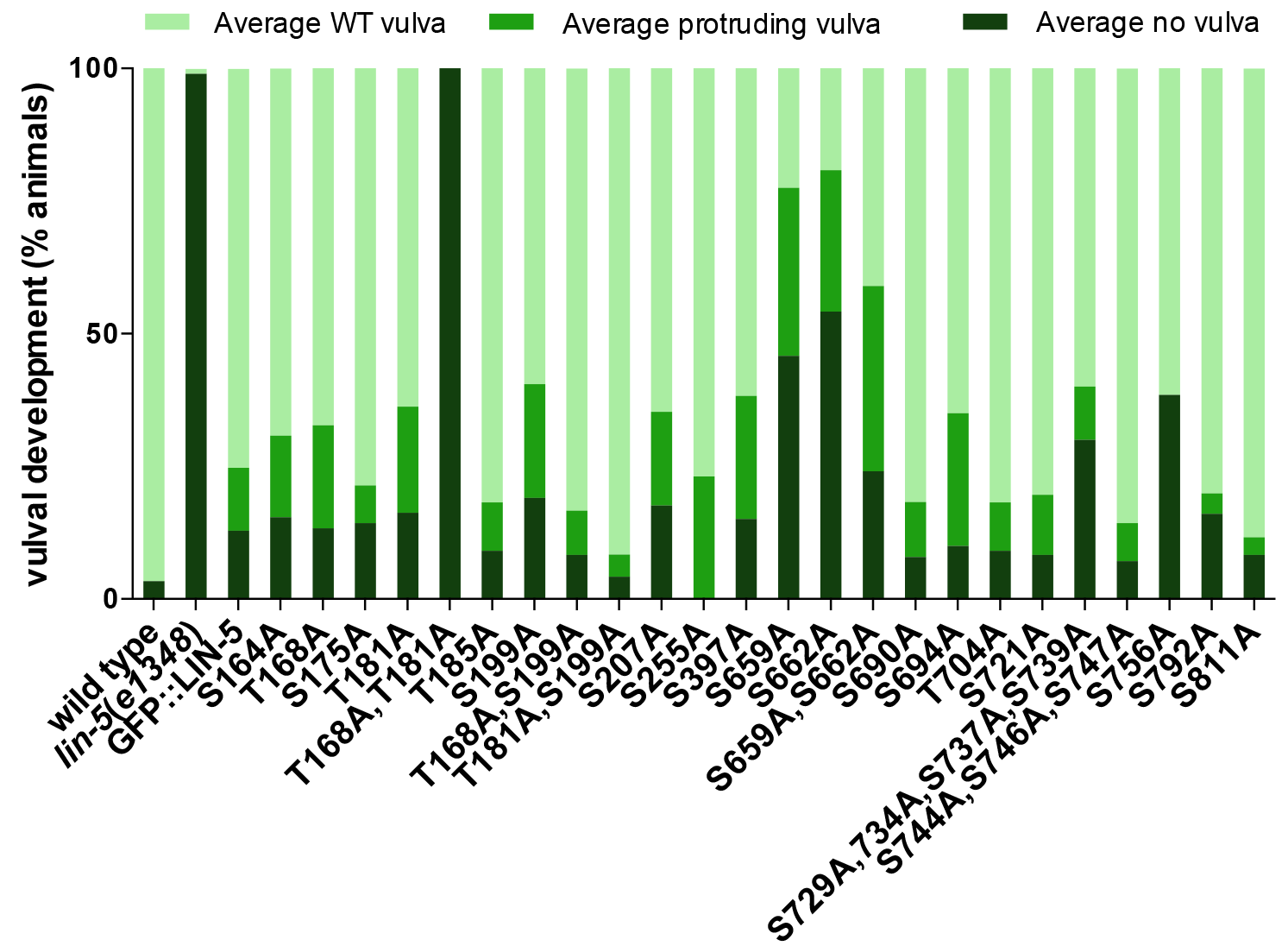

Supplement: S1 Fig — Quantification of vulval development in heterozygous lin-5(e1348) / mIn1 (Wild type), homozygous lin-5(e1348) animals, and homozygous lin-5(e1348) animals expressing gfp::lin-5 transgenes. (TIF) [file pgen.1006291.s001.tif]

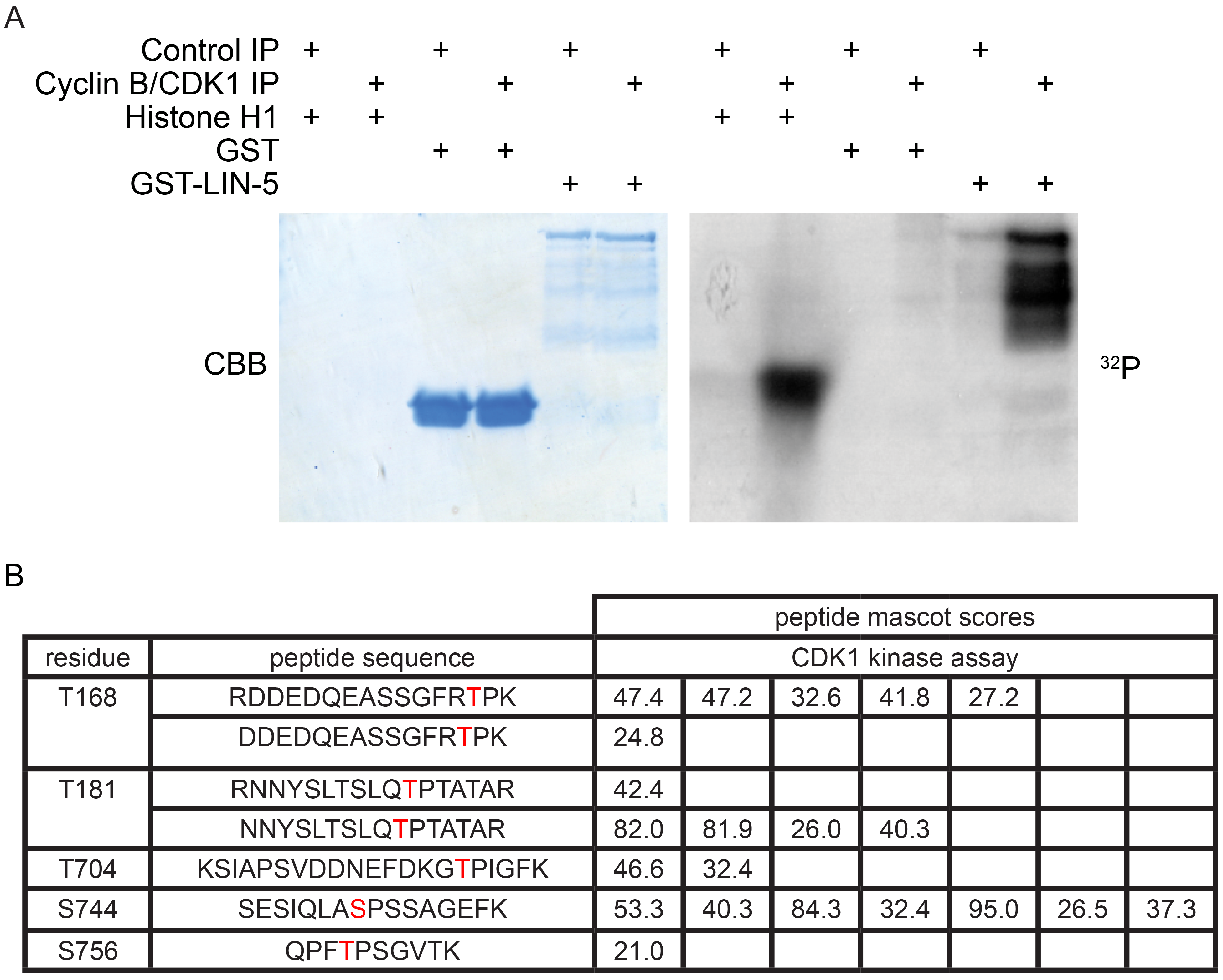

Supplement: S2 Fig — (A) In vitro CDK1 kinase assay with recombinant GST-LIN-5, GST alone, or Histone H1. Left; coomassie brilliant blue (CBB)-stained gel. Right; autoradiogram. (B) List of LIN-5 phosphopeptides identified by mass spectrometry analysis of CDK1 in vitro kinase assay with GST-LIN-5. Phosphopeptides are shown with individual Mascot Scores. Only Mascot Scores above 20 were accepted as reliable peptide identifications. (TIF) [file pgen.1006291.s002.tif]

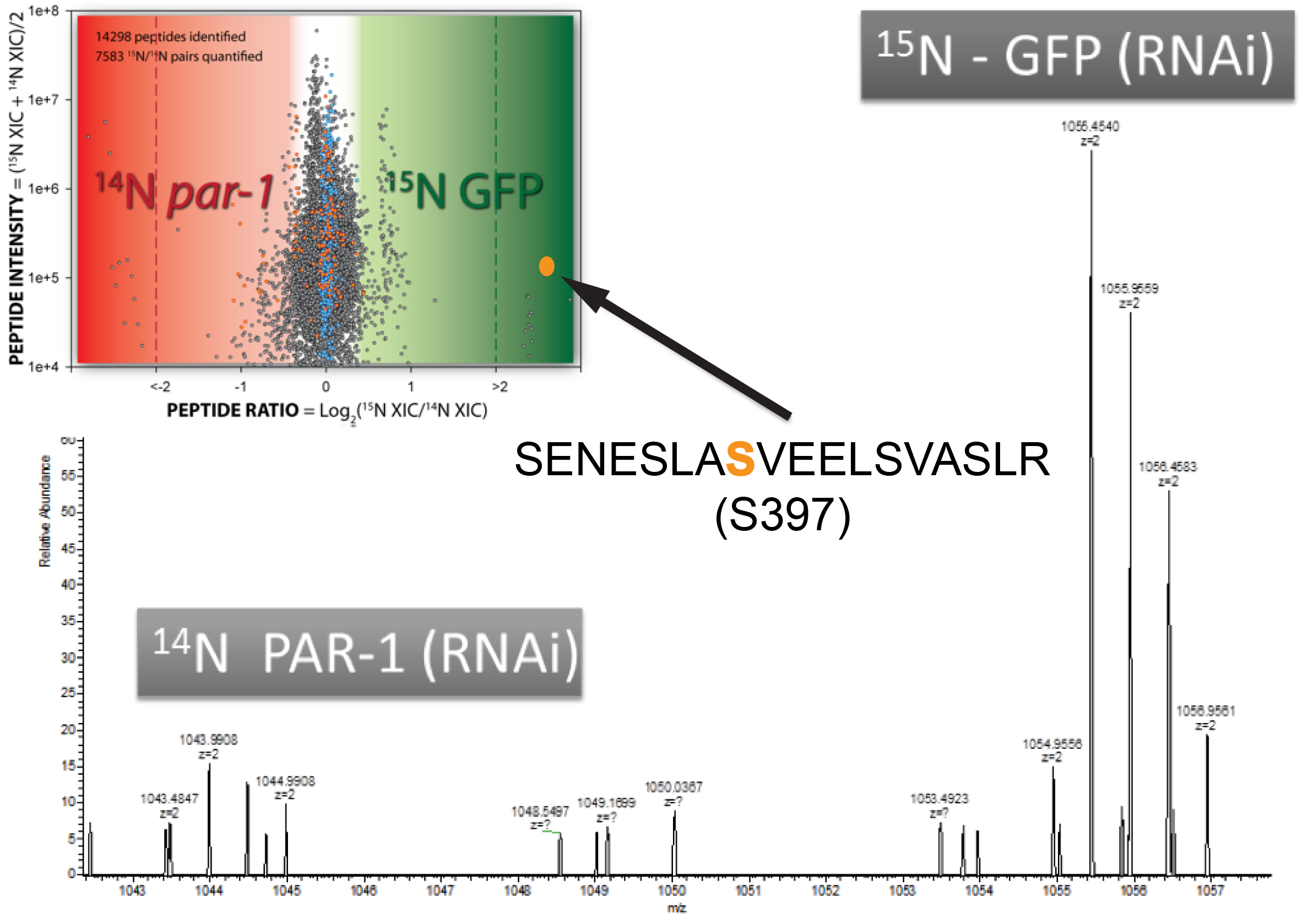

Supplement: S3 Fig — Log2 ratios for all of the quantified 15N/14N peptide pairs as a function of their mass-spectrometry intensities in the three LIN-5 immunoprecipitates. LIN-5 phosphopeptides are represented in red, and LIN-5 regular peptides are represented in blue. Peptides belonging to other proteins are shown in grey. Peptide intensities were calculated using the average of the 14N and 15N extracted ion chromatograms. (TIF) [file pgen.1006291.s003.tif]

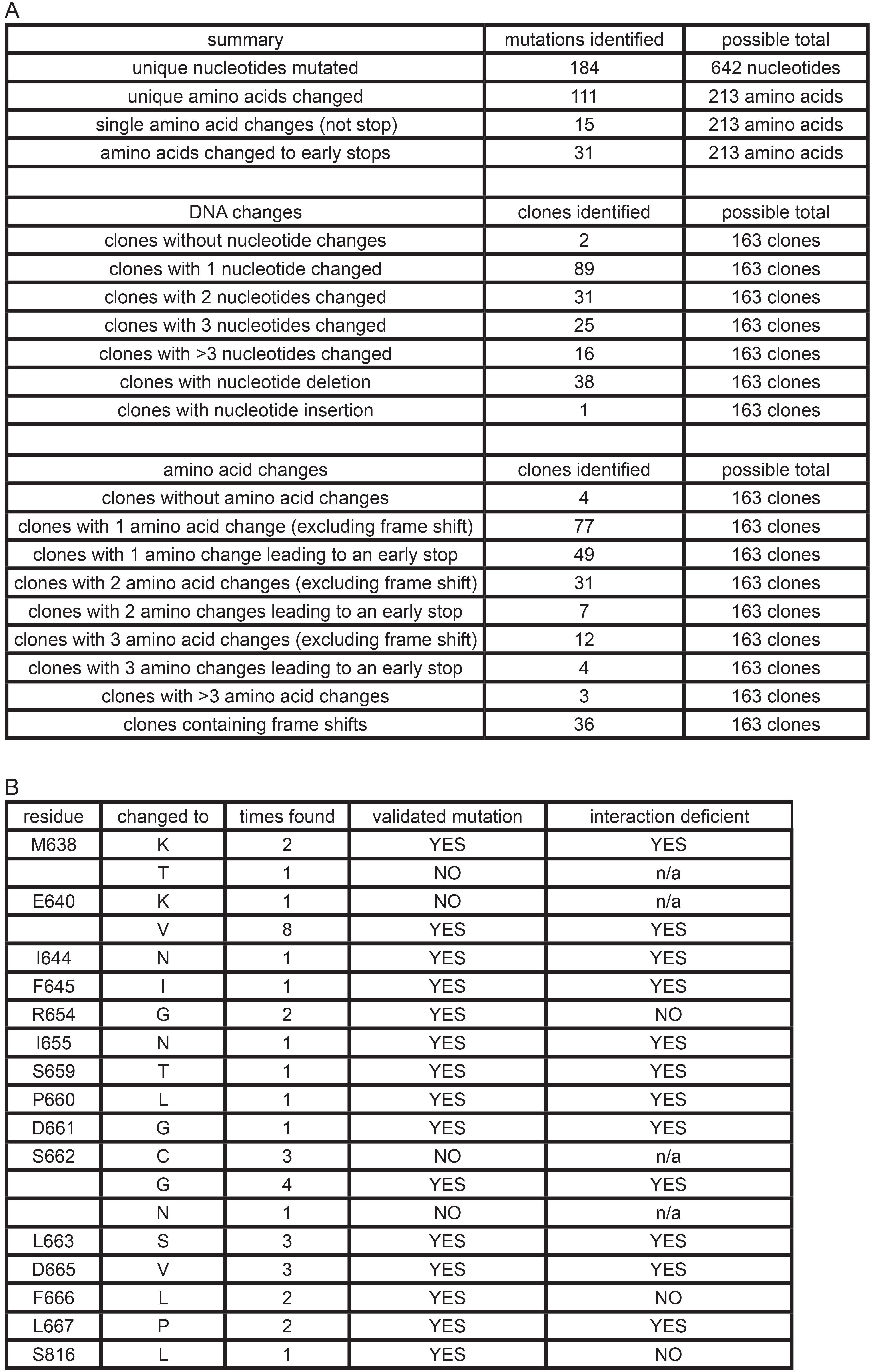

Supplement: S4 Fig — (A) Summary of sequencing results of all interaction-deficient alleles of LIN-5 identified in the reverse yeast two-hybrid assay before further validation. (B) Overview of all interaction deficient alleles of LIN-5 containing a single amino acid change identified in the reverse yeast two-hybrid assay before further validation. Validation is shown in Fig 3B. (TIF) [file pgen.1006291.s004.tif]

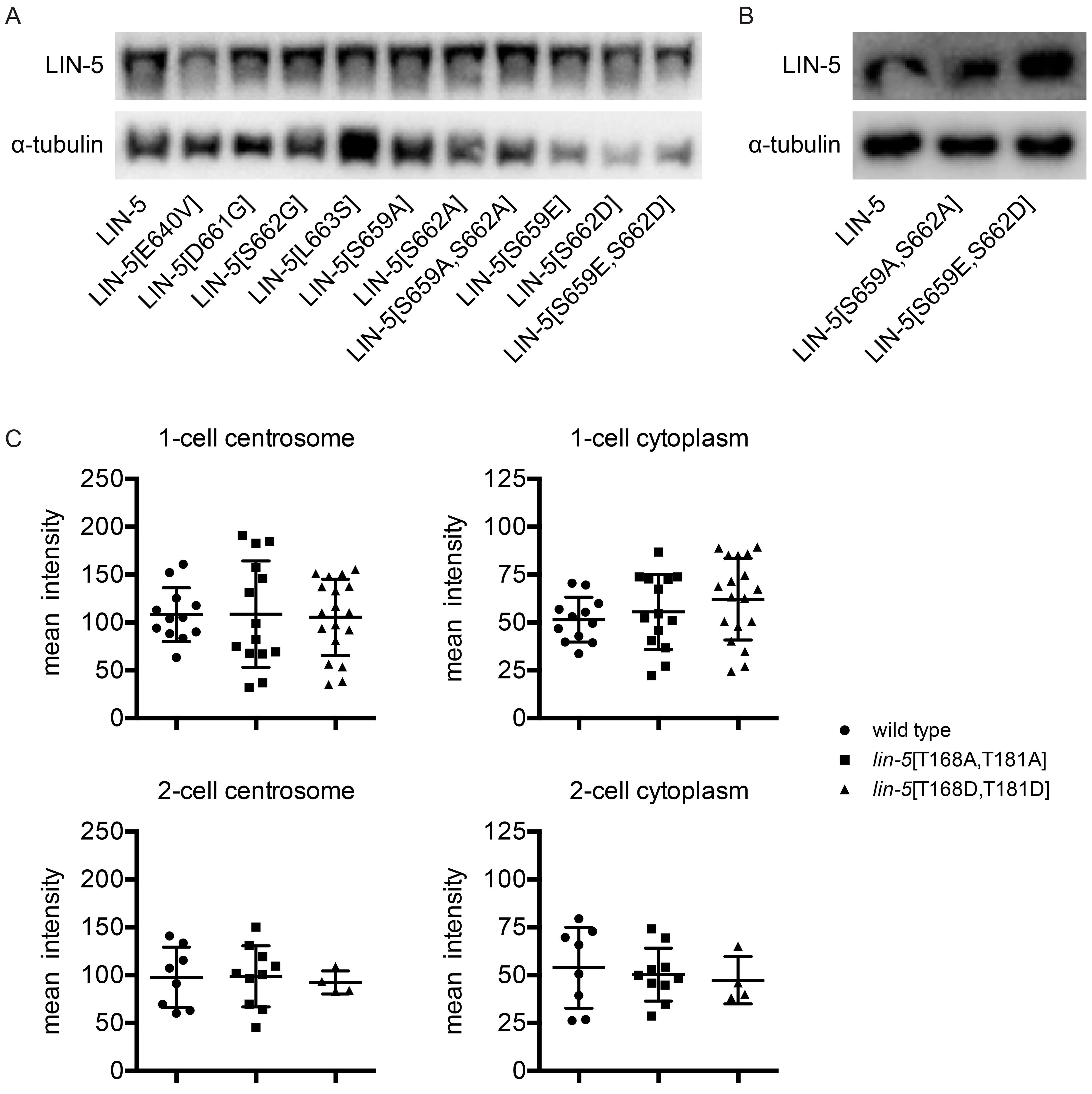

Supplement: S5 Fig — (A) Western blots of lysates of yeast clones containing the indicated LIN-5 expression constructs, probed for LIN-5 and tubulin (loading control) levels. (B) Western blots of C. elegans lysates with detection of LIN-5 and tubulin (loading control). (C) Quantification of mean intensity of immunostainings of lin-5[mutant T168,T181] / egfp::lin-5 embryos treated with egfp RNAi and stained with LIN-5 antibodies. Graphs indicate single values for centrosomes and cytoplasm in one- and two-cell embryos. (TIF) [file pgen.1006291.s005.tif]

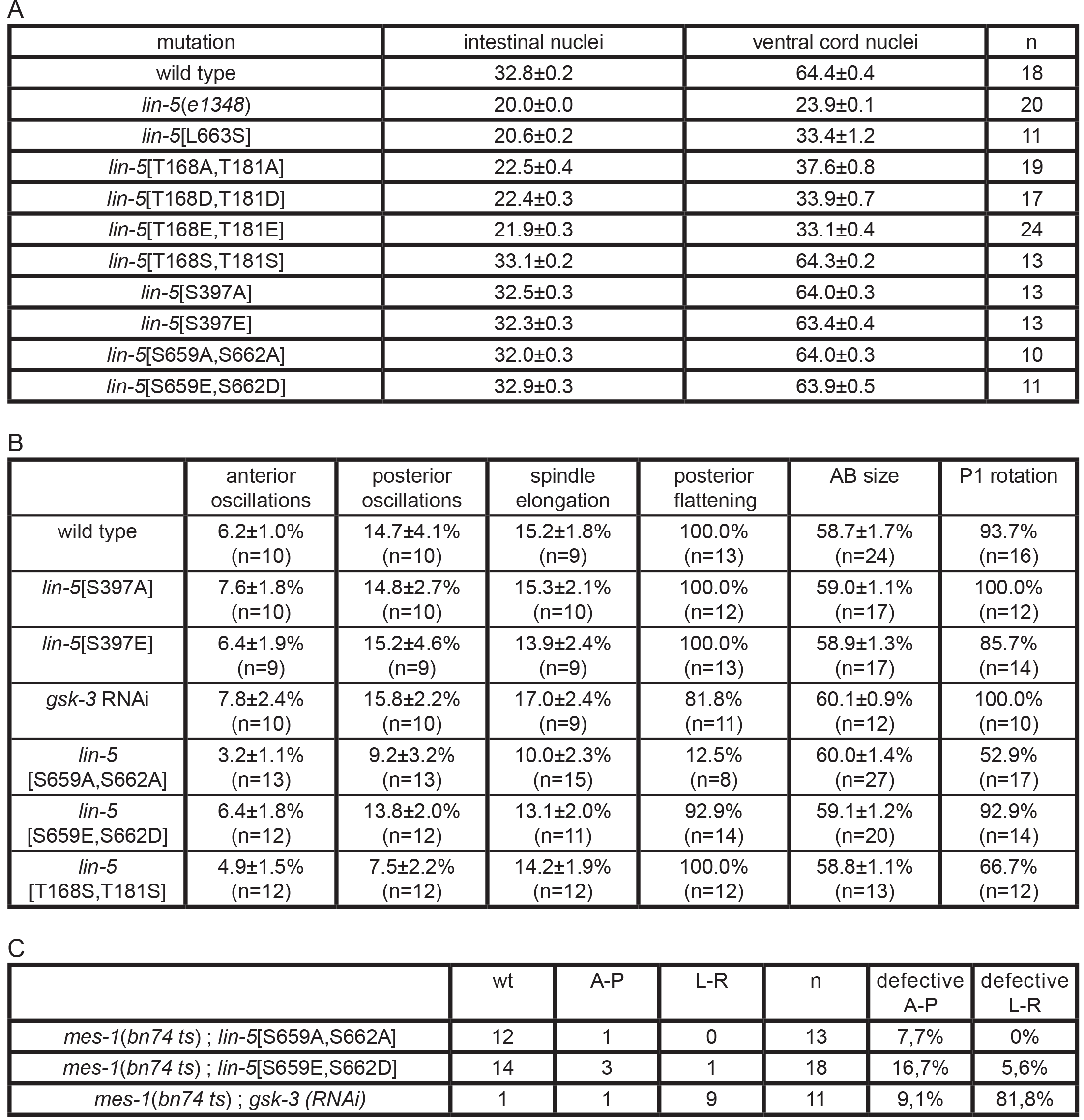

Supplement: S6 Fig — (A) Averages of quantification of intestinal nuclei and P-cells plus juvenile motor neurons (P2-P10 region) by propidium iodide staining in wild type, homozygous LIN-5 phosphorylation mutants, and homozygous lin-5(e1348) null animals. Statistical analysis in s.e.m., analyzed by Graphpad PRISM. (B) Statistical averages of DIC microscopy imaging of hallmarks of the first 2 embryonic divisions in LIN-5 phosphorylation mutants. Oscillations are plotted in percentage of embryo height, elongation and AB size as a percentage of embryo width, flattening and rotation as a total fraction of analyzed embryos. Statistical analysis in s.e.m., analyzed by Graphpad PRISM. (C) Quantification of EMS spindle rotation in mes-1(bn74 ts); lin-5[S659,S662] phosphorylation mutant and mes-1(bn74 ts); gsk-3(RNAi) embryos. Spindle rotation was quantified by live-imaging of the gfp::tubulin marker. Wt indicates number of embryos with wild type rotation, defective A-P indicates number of embryos with a failure to fully align in the anterior-posterior direction, defective L-R indicates number of embryos with a failure to rotate in the left-right direction. (TIF) [file pgen.1006291.s006.tif]

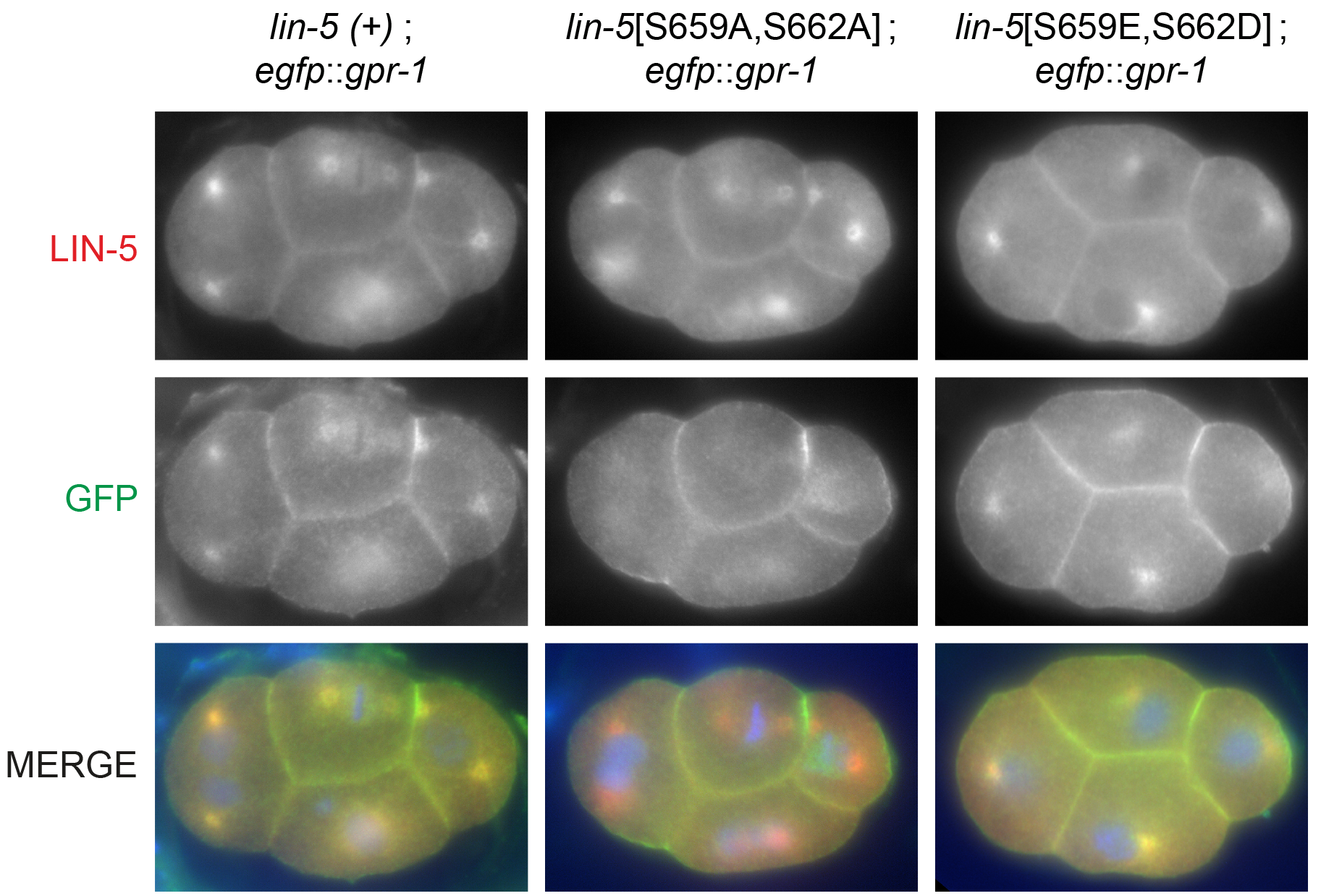

Supplement: S7 Fig — Immunohistochemical staining of embryos expressing wild type or phosphomutant lin-5 and endogenously tagged egfp::gpr-1. Representative images of four-cell embryos, stained with anti-LIN-5 (red) and anti-eGFP (green) antibodies, and DAPI to visualize DNA. All images taken with same exposure time, objective and magnification. Anterior to the left, ventral up. (TIF) [file pgen.1006291.s007.tif]

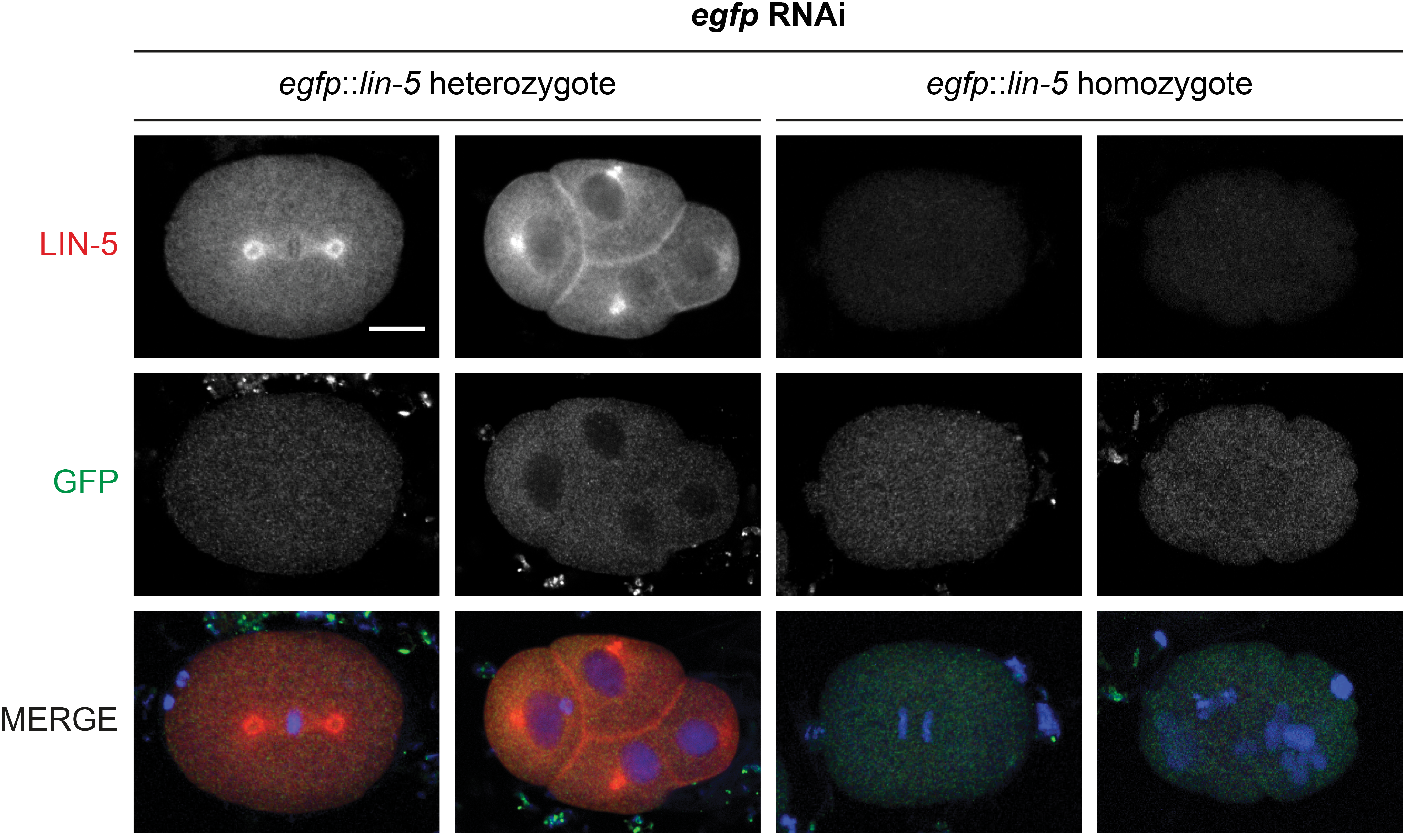

Supplement: S8 Fig — Immunohistochemical staining of heterozygous and homozygous egfp::lin-5 C. elegans embryos with anti-LIN-5 (red) and anti-GFP (green) antibodies, DNA stained with DAPI. Two representative embryos are shown for every condition. All images same objective and magnification, anterior to the left, scale bars 10 μm. (TIF) [file pgen.1006291.s008.tif]

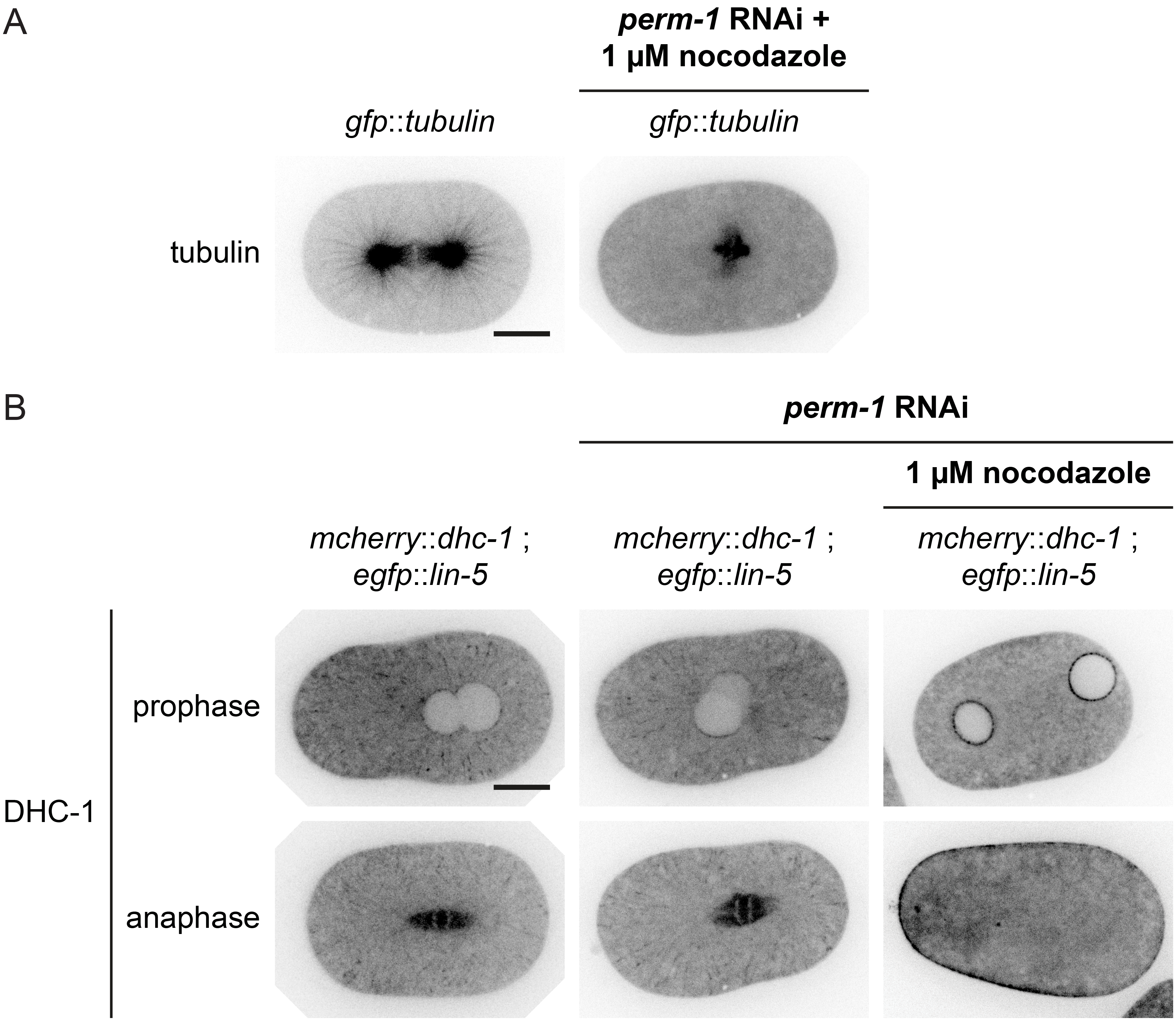

Supplement: S9 Fig — (A) Representative snapshots of live imaging of GFP::tubulin in gfp::tubulin one-cell embryos treated with or without perm-1 RNAi + 1 μM nocodazole, and imaged by spinning disk confocal microscopy. Scale bars, 10 μm, all images with same objective and magnification. (B) Representative snapshots of live imaging of mCherry::DHC-1 in mcherry::dhc-1; egfp::lin-5 one-cell embryos in prophase and metaphase treated with or without perm-1 RNAi in the presence or absence of 1 μM nocodazole, and imaged by spinning disk confocal microscopy. All images taken with same objective and magnification, anterior to the left, scale bars 10 μm. (TIF) [file pgen.1006291.s009.tif]
