# Supplementary material for: Multisite Phosphorylation of NuMA-Related LIN-5 Controls Mitotic Spindle Positioning in C. elegans
Source: PLoS Genet. 2016 Oct 6;12(10):e1006291. doi: 10.1371/journal.pgen.1006291 (PMC5053539; doi:10.1371/journal.pgen.1006291)
Supplement: S1 Table — Overview of all strains used in this study, including the corresponding figure. CRISPR/Cas9 genome engineered strains are indicated in the comment section. (PDF) [file pgen.1006291.s031.pdf]

Table S1. Overview of *C. elegans* strains used in this study

Strain	genotype	comment
N2	wild type	Fig. 4A-C, S6A-B
SV13	*lin-5*(*e1348*) / *mnC1 dpy-10*(*e128*) *unc-52*(*e444*)	Fig. 4A-B, S6A
SV918	*lin-5*(*e1348*) / *mIn1*	Fig. 1, S1
SV1568	*lin-5(he237*[S659A,S662A])	Fig. 4A, S6A-B, CRISPR allele
SV1569	*gpr-1(he238[fkbp:: egfp::gpr-1]*)	Fig. 5, CRISPR allele
SV1585	*lin-5(he237*[S659A,S662A]) ; *ruIs57* [*unc-119*(+) ; *ppie-1::gfp::β-tubulin*]	Fig. 4D
SV1586	*lin-5(he240*[L663S])	Fig. 4A, S6A, CRISPR allele
SV1587	*lin-5(he241*[T168A,T181A]) / +	CRISPR allele
SV1588	*lin-5(he242*[S397E])	Fig. 4A, S6A-B, CRISPR allele
SV1589	*lin-5(he244[egfp::lin-5])*	Fig. S7, CRISPR allele
SV1590	*lin-5(he243*[S397A])	Fig. 4A, S6A-B, CRISPR allele
SV1594	*lin-5(he242*[S397E]) ; *ruIs57[unc-119(+) ; ppie-1::gfp::β-tubulin]*	Fig. 4D
SV1596	*lin-5(he243*[S397A]) ; *ruIs57[unc-119(+) ; ppie-1::gfp::β-tubulin]*	Fig. 4D
SV1600	*lin-5(he249*[S659E,S662D])	Fig. 4A, S6A-B, CRISPR allele
SV1618	*lin-5(he249*[S659E,S662D]) ; *ruIs57[unc-119(+) ; ppie-1::gfp::β-tubulin]*	Fig. 4D
SV1619	*dhc-1(he250[mcherry::dhc-1])*	Fig. 7A, S8B, CRISPR allele
SV1620	*lin-5(he251*[T168D,T181D]) / +	CRISPR allele
SV1621	*lin-5(he251*[T168D,T181D]) / *lin-5(he244[egfp::lin-5])*	Fig. 4A, 6, S6A
SV1622	*lin-5(he241*[T168A,T181A]) / *lin-5(he244[egfp::lin-5])*	Fig. 4A, 6,S6A
SV1635	*dhc-1(he250[mcherry::dhc-1])* ; *lin-5(he244[egfp::lin-5])*	Fig. 7A
SV1638	*dhc-1(he250[mcherry::dhc-1])* ; *lin-5(he251*[T168D,T181D]) / *lin-5(he244[egfp::lin-5])*	Fig. 7A
SV1639	*dhc-1(he250[mcherry::dhc-1])* ; *lin-5(he241*[T168A,T181A]) / *lin-5(he244[egfp::lin-5])*	Fig. 7A
SV1663	*lin-5(he237*[S659A,S662A]) ; *gpr-1(he238[fkbp::egfp::gpr-1])*	Fig. 5
SV1664	*lin-5(he249*[S659E,S662D]) ; *gpr-1(he238[fkbp:: egfp::gpr-1])*	Fig. 5
SV1695	*lin-5(he261*[T168E,T181E]) / +	Fig. 4A, S6A, CRISPR allele
SV1700	*lin-5(he241*[T168A,T181A]) / *lin-5(he244[egfp::lin-5])* ; *ruIs57[unc-119(+) ; ppie-1::gfp::β-tubulin]*	Fig. 4E
SV1701	*lin-5(he251*[T168D,T181D]) / *lin-5(he244[egfp::lin-5])* ; *ruIs57[unc-119(+) ;*	Fig. 4E

	ppie-1::gfp::β-tubulin]	
SV1702	*dhc-1(he250[mcherry::dhc-1])* ; *ruIs57[unc-119(+)ppie-1::gfp::β-tubulin]*	Fig. 7B
SV1783	*lin-5(he237*[S659A,S662A]) ; *ruIs57[unc-119(+) ; ppie-1::gfp::β-tubulin]* ; *mes-1(bn74*)	Fig. S6C, maintained at 15°C
SV1784	*lin-5(he249*[S659E,S662D]) ; *ruIs57[unc-119(+) ; ppie-1::gfp::β-tubulin]* ; *mes*-1(*bn74*)	Fig. S6C, maintained at 15°C
SV1785	*ruIs57[unc-119(+) ; ppie-1::β-tubulin::GFP]* ; *mes-1(bn74*)	Fig. S6C, maintained at 15°C
SV1898	*dhc-1(he250[mcherry::dhc-1])* ; *lin-5(he241*[T168A,T181A]) / *lin-5(he244[egfp::lin-5])* ; *ruIs57[unc-119(+) ; ppie-1::gfp::β-tubulin]*	Fig. 7B
SV1899	*dhc-1(he250[mcherry::dhc-1])* ; *lin-5(he251*[T168D,T181D]) / *lin-5(he244[egfp::lin-5])* ; *ruIs57[unc-119(+) ; ppie-1::gfp::β-tubulin]*	Fig. 7B
SV1901	*lin-5* (*he281*[T168S,T18S])	Fig. 4A, S6A
SV1902	*dhc-1(he250[mcherry::dhc-1])* ; *lin-5(he244[egfp::lin-5])* ; *ruIs57[unc-119(+) ; ppie-1::gfp::β-tubulin]*	Fig. 7B, CRISPR allele
SV1911	*lin-5* (*he281*[T168S,T18S]) ; *ruIs57[unc-119(+) ; ppie-1::gfp::β-tubulin]*	Fig. 4E
AZ244	*unc-119 (ed3)* ; *ruIs57[unc-119(+) ; ppie-1::gfp::β-tubulin]*	Fig. 4D-E, S8A
SS392	*mes-1(bn74*)	maintained at 15°C

Overview of all strains used in this study, including the corresponding figure. CRISPR/Cas9 genome engineered strains are indicated in the comment section.
